# CaSPIAN: A Causal Compressive Sensing Algorithm for Discovering Directed Interactions in Gene Networks

**DOI:** 10.1371/journal.pone.0090781

**Published:** 2014-03-12

**Authors:** Amin Emad, Olgica Milenkovic

**Affiliations:** Department of Electrical and Computer Engineering, University of Illinois at Urbana-Champaign, Urbana, Illinois, United States of America; Universiteit Gent, Belgium

## Abstract

We introduce a novel algorithm for inference of causal gene interactions, termed CaSPIAN (Causal Subspace Pursuit for Inference and Analysis of Networks), which is based on coupling compressive sensing and Granger causality techniques. The core of the approach is to discover sparse linear dependencies between shifted time series of gene expressions using a sequential list-version of the subspace pursuit reconstruction algorithm and to estimate the direction of gene interactions via Granger-type elimination. The method is conceptually simple and computationally efficient, and it allows for dealing with noisy measurements. Its performance as a stand-alone platform without biological side-information was tested on simulated networks, on the synthetic IRMA network in *Saccharomyces cerevisiae*, and on data pertaining to the human *HeLa* cell network and the SOS network in *E. coli*. The results produced by CaSPIAN are compared to the results of several related algorithms, demonstrating significant improvements in inference accuracy of documented interactions. These findings highlight the importance of Granger causality techniques for reducing the number of false-positives, as well as the influence of noise and sampling period on the accuracy of the estimates. In addition, the performance of the method was tested in conjunction with biological side information of the form of sparse “scaffold networks”, to which new edges were added using available RNA-seq or microarray data. These biological priors aid in increasing the sensitivity and precision of the algorithm in the small sample regime.

## Introduction

One of the unresolved open problems in systems biology is discovering causal relationships among different components of biological systems. Gene regulatory networks, protein-protein interaction networks, chemical signaling and metabolic networks are all governed by causal relationships between their agents that determine their functional roles. Discovering causal relationships through experiments is a daunting task due to the technical precision and output volumes required from the experiments and due to the large number of interconnected and dynamically varying components of the system. It is therefore of great importance to develop a precise analytical framework for quantifying causal connections between genes in order to elucidate the gene interactome based on limited and noisy experimental data. Statistically inferred interactions may be used to guide the experimental design process, helping with further refinement of the modeling framework [1], [2]. Unfortunately, to date most reverse engineering algorithms have offered very few reliable outcomes for even moderately sized networks and were hardly ever experimentally tested – these and other shortcomings of existing inference techniques and models were described in detail in [3]. Consequently, algorithmic developments are focusing on small network components of *prokaryotic* or simple *eukaryotic* cell lines and on the more conservative – yet reliable – task of identifying a small number of highly accurate causal links.

One way to detect if a gene causally influences another gene is to monitor if changes in the expression of the potential regulator gene affect the expression of the target gene in the presence of at least one additional network component [4], [5]. This type of analysis is frequently used in combination with expression clustering and classification techniques [6]. A number of authors also suggested the use of Bayesian networks [7]–[11], Boolean networks [12], [13], differential equations [13], [14], stochastic networks [15], [16], finite state linear models [17] and other machine learning tools for network inference. Algebraic techniques were described in [18], [19], while different information-theoretic approaches were proposed in [20]–[27]. For an overview of a variety of other methods for reverse engineering of gene regulatory networks, the interested reader is referred to [28]–[35].

Sparsity of gene regulatory networks was exploited in a number of different inference frameworks, including transcription factor interaction analysis [2], [36]–[38]. Most of the proposed methods integrate sparsity priors through a form of Lasso penalty [39]. The algorithms reduce to an optimization problem that in its simplest form tries to minimize an objective function consisting of two terms: the first term is the 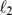 norm of the reconstruction error, while the second term is a regularization term, equal to the 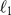 norm of the sought solution. The main difficulties associated with the Lasso framework are solving a high-dimensional optimization problem and properly choosing the coefficient of the regularization term(s). In most cases, the regularization coefficient is either chosen heuristically or using an optimization procedure which increases the complexity of the algorithm without providing provable performance guarantees. Parameter tuning issues also make the comparison of results generated by Lasso for different objective functions hard to accomplish in a fair manner.

An alternative to the Lasso approach is a greedy compressive sensing framework, which overcomes some of the shortcomings of Lasso while still utilizing the sparsity of the network. Compressive sensing (CS) is a dimensionality reduction technique with widespread applications in signal processing and optimization theory [40], [41]. CS allows for inferring sparse structures given a small number of linear measurements, usually generated in a random fashion. As such, it naturally lends itself for use in biological inference problems involving sparse interaction networks.

Motivated by recent advances in CS theory and its application in practice, we introduce the concept of *causal compressive sensing* and design new greedy list-reconstruction algorithms for inference of causal gene interactions; as part of the process, we generate two sparse models for each potential interaction pattern and infer causality by comparing the residual errors of the models using statistical methods. Furthermore, in CS, the most difficult task consists of finding the support (i.e. the nonzero entries) of a sparse signal. This is accomplished by inferring the subspace in which the vector of observation lies. As a result, the complicated process of choosing the regularization coefficient in Lasso is substituted by the more natural task of choosing a “consistency” level between the vector of observations and its representation in the estimated subspace.

The CS approach has not been widely used for gene regulatory network inference; to the best of the author's knowledge, only the methods in [42] and [43] described compressive sensing algorithms for linear models. Both papers deal with non-causal inference. In our work, we propose a method for identifying causal gene interactions based on a combination of two ideas: greedy CS reconstruction and Granger causality, or elimination analysis. The CS model is motivated by a technique for face recognition used in computer vision, first described in [44]. The crux of the approach is to efficiently find a sparse linear representation of an image of one individual in terms of images of that and other individuals, taken under many different conditions. One component of the setup is reminiscent to the method described in [43], where expression levels of genes taken under different experimental conditions (or under different gene knockout scenarios) are represented as vectors for which a sparse representation is sought. However, the results in [43] are based on an 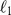 optimization method and only infer *non-causal* interaction among the genes. In addition, CS was used only as a preprocessing step; the obtained CS results were combined with extensive prior biological information, and the gene interactions were inferred through supervised learning performed by AdaBoost. It is worth mentioning that AdaBoost and similar boosters are highly susceptible to random classification noise, thereby limiting their applications in biological data analysis [45].

In this manuscript, we propose two causal CS inference approaches. In order to infer causality, we apply these approaches to two different combinations of gene expression profiles shifted in time, one of which contains a potential regulator and another, which does not contain a potential regulator. Each dataset gives rise to a certain representation error, and one may infer the level of influence of genes on each other based on the differences in the representation errors and by using the F-test [46]. The first method is an unsupervised learning scheme and therefore has the advantage that it does not need to be combined with learning steps involving biological side-information in order to produce good predictions. In the second method, in order to incorporate biological priors into the subspace pursuit process, we propose using an experimentally verified “scaffold network”. This network consists of a small number of highly reliable edges, chosen based on the number of times they were reported in the literature, the number of different experimental methods used to verify them and similar considerations. The use of a scaffold network may resolve some ambiguities in the subspace selection process, which often lead to inference errors and hence improve the overall performance of causal CS methods.

At the core of our computational method is the subspace pursuit (SP) algorithm, which we described in [47]. We adapt this greedy approach into an algorithm termed list-SP. List-SP sequentially scans subspaces of measurements with different dimensions and creates an output that consists of the union of basis vectors for all identified subspaces. An advantage of the proposed algorithms is that one can take advantage of the prior information on the sparsity of the network, i.e. the in-degree of the nodes. If such information is not available, a rough estimate or an upper bound on the in-degree of the nodes is sufficient. In particular, the upper bound can be chosen large enough to ensure that it exceeds the largest in-degree of the network, and then the false-positives can be rejected throughout the F-test step of the inference algorithm.

The main finding of our analysis indicates that causal compressive sensing can infer a relatively large fraction of causal gene interactions with very small false-positive rates when applied to small and moderate size networks. This finding is supported by simulated data, synthetic data from the IRMA network in *Saccharomyces cerevisiae* [48], and biological data from the human HeLa cell network and the SOS network of *E. coli* [49]. The success probability is, as expected, highly influenced by the noise variance of the experiment and by the sampling time of the expressions. Our analysis of these phenomena adds to the understanding of the limitations of causal inference under imperfect measurement conditions, as well as the role of biological side information in reducing inference error rates. It also explains why available methods may not result in an improved detection probability upon adding as many time-shifted expression profiles as available, since gene expressions are usually measured at too widely separated times and have different time periods between the measurements.

## Methods

### Compressive Sensing

Compressive sensing (CS) is a technique for sampling and reconstructing sparse signals, i.e. signals that can be represented by 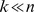 significant coefficients over an 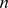-dimensional basis. What distinguishes CS from other dimensionality reduction techniques is that it operates with a small number of measurements [40], [41] that allow for polynomial-time reconstruction of the sparse signal.

Assume that one is interested in finding a vector 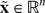 using a (noisy) observation 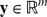 obtained according to 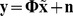, for a known sensing matrix 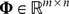, with 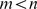; here, 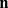 denotes the noise vector. In general, the problem cannot be solved uniquely. However, if 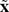 is 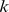-sparse, i.e., if it has up to 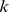 nonzero entries, one may recover 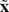 uniquely if 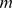 is large enough. This can be achieved by finding the sparsest signal consistent with the vector of measurements [40], i.e.
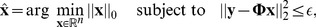
(1)where 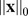 denotes the 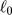 norm of 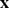 (i.e., the number of non-zero entries of 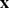), while 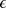 denotes a parameter that depends on the level of measurement noise. It can be shown that the 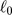 minimization method can exactly reconstruct the original signal in the absence of noise using a properly chosen sensing matrix 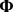 whenever 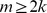. However, 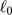 minimization is a computationally hard combinatorial problem and cannot be performed efficiently.

On the other hand, it is known that an 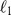 convex relaxation of (1) can accurately approximate the signal 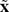 in polynomial time if 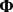 satisfies the so-called restricted isometry property (RIP) and provided that 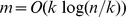 [40], [41]. This optimization problem may be stated as
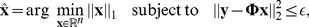
(2)where, as before, 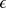 depends on the noise variance. One should note that the 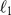 minimization in Equation (2) is closely related to the previously mentioned Lasso problem [39]

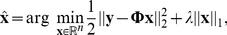
(3)where 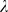 is a regularization parameter. If 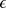 and 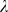 in Equations (2) and (3) satisfy some special conditions, the two problems are equivalent; however, characterizing the relationships between 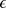 and 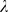 is difficult, except for the special case of orthogonal sensing matrices 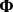 [54]. In most other cases, it is easier and more natural to find an appropriate value of 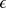 than 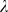 [51], since 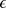 is proportional to the noise variance. As a result, the CS framework eliminates the computational issues of Lasso regarding parameter selection.

Although the 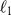 relaxation can be reformulated as a linear program (LP), for high-dimensional vectors it is desirable to use greedy algorithms as they offer significant reductions in computational complexity while ensuring performance comparable to that of LP methods. Another advantage of greedy methods is the ease of adding constraints and adapting the method to the problem at hand. For an in-depth discussion of one such greedy algorithm, Subspace Pursuit (SP), the interested reader is referred to [47].

### The List-SP algorithm

We next introduce the List-SP method, a modification of the SP algorithm [47] that is designed to increase the number of true positives found using SP while preserving the benefits of SP (including low complexity, ability to incorporate side information on the in-degree of the nodes, etc.).

Assume that for a vector 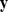, a sensing matrix 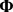, and a fixed value of 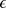, one is interested in finding a 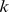-sparse estimate of 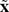, denoted by 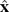, such that 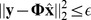. The main challenge is to find the support of 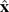, or the columns of 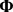 indexed by 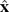; given the support, the values of the non-zero entries may be obtained via pseudo-inversion [52]. List-SP is an iterative algorithm for solving this problem in 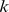 iterations. In the 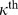 iteration, 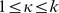, a set of 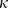 columns of 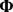 is identified, such that the linear combination of these columns represents 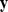 with smallest possible error. The union of the sets of columns found during these 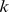 iterations forms a subspace that with high probability contains the subspace spanned by the columns of 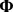 indexed by 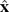. In order to find the set of columns in the 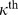 iteration, SP first finds a set of 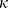 columns of the sensing matrix with highest correlation with 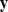; then, iteratively, the SP algorithm tests groups of columns in a greedy manner to augment the existing set of 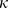 columns of 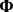 with 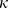 additional columns that together most likely span the subspace in which 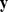 lies. Upon finding such a set, SP updates the list of columns by discarding the 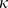 “least reliable” of them. The procedure continues until a desired accuracy is achieved [47]. Note that the reason for forming the union of subspaces in list-SP is that it may happen that some of the recovered columns for sparsity level 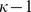 are not present among the recovered columns for sparsity level 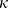. By combining the results obtained using SP for different values of 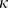, we reduce the chance of missing an important column of 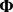, given that we do not know the sparsity level. In the context of gene interaction inference, we effectively reduce the probability of false-negatives. This significantly improves the performance of the List-SP compared to SP with respect to false-negatives, as we demonstrate in the results section. Note that the computational complexity of List-SP algorithm is 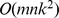, compared to the complexity of the classical SP, which equals 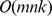 [47].

### Granger Causality

Only a few algorithms using signal sparsity were successfully integrated into causal inference models [2], [36]. One such causality testing scheme, originally proposed in econometrics, is Granger causality [53]. In its original incarnation, Granger causality was presented as a heuristic statistical concept based on prediction. The method has the goal to determine if a time series of past observations of a process helps to predict the future values of another process. Granger causality only considers two stochastic stationary processes, in addition to an auxiliary process required as a “causal reference” [5]. An extension of this definition to more than two stationary processes, dubbed conditional Granger causality, was introduced in [54]. In the context of linear regression, this causal model may be described as follows. Assume that the value of the process 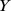 at time 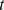 can be predicted using the values of the processes 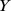 and 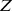 at 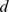 past time-points through the coefficients 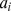 and 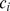, 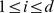. The *restricted model* takes the form
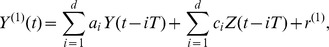
where 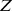 is the reference process, and 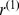 represents the approximation residual. Augmenting this model with the 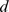 past values of 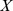 yields the *unrestricted model*

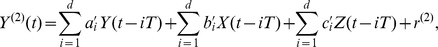
where 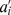, 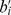, and 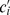, 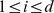, are model coefficients, and 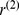 represents the approximation residual. If the variance of 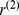 is “significantly” smaller than the variance of 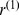 in a suitable statistical framework, then 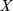 Granger-causes 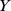 conditioned on 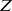, which we denote by 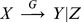.

In most cases, causality and co-integration are assessed via Augmented Dickey Fuller (ADF) tests or F-tests on the residual. In a nutshell, these tests determine the significance of the change in the value of the variance of the residuals [46]. Assume that one is given different realizations of the processes 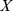, 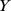, and 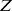. These realizations can be used to form vector time-series models of the restricted and unrestricted models. To determine if 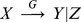, we assume the null hypothesis that 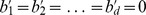. The F-statistic for this null hypothesis is
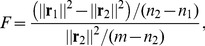
(4)where 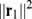 and 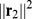 are the squared norms of the residuals in the restricted model and the unrestricted model, respectively; 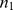 and 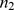 are the total number of parameters in the restricted and unrestricted model, respectively, and 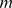 is the total number of realizations of the processes at each time point. Under the null hypothesis, the F-statistic follows an F-distribution with 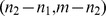 degrees of freedom [46]. The null hypothesis is rejected if the F-statistic is greater than a critical value, calculated using the F-distribution for a desired significance level, 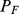.

In what follows, the CS-Granger causality approach is applied on gene expression data sampled at a small number of time instances. There are two main issues to be addressed in this context: how to discover linear relationships between expression profiles that may be (and usually are) correlated with each other and how to adapt the sensing matrix 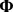 to perform meaningful Granger-type tests. The processes of interest, 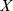, 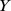 and 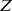 represent the regulated gene, the regulator gene and a *collection of candidate genes*, which contain both genes that causally influence 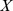 (excluding 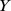) and genes that do not causally influence 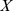. In other words, 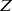 is a vector random process 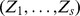, where 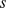 denotes the number of candidate genes.

### The CaSPIAN algorithm for gene network inference

In order to combine CS techniques, in particular List-SP, with Granger causality, we assume that the gene expressions may be modeled via a linear regression. In addition, we assume that different realizations of the model are available through different experiments. These realizations are used to form a vector regression model for the gene expressions. More precisely, assume that one is interested in finding the directed graph corresponding to causal relationships of 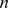 genes, denoted by 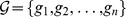, using gene expression levels obtained under different experimental conditions. If the structure of the gene regulatory network does not change significantly under these conditions, one should be able to form expression profiles for each gene by concatenating the expression levels in different experiments. In particular, conditions that do not affect a subnetwork of the gene regulatory network can be used to infer the causal relationships among the genes of the subnetwork of interest. The procedure of forming the gene expression profiles is described in more detail at the end of this section.

Let 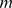 denote the number of experiments and assume that in each experiment the expression level of each gene is given for 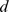 time points. Let 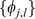 denote the set of expression profiles of genes, where 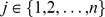 and 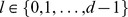; 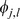 is a column-vector denoting the expression profile of 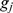 corresponding to time 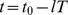, where 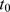 is the latest available time-point and 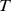 is the sampling period between the time-points. Assume that one is interested in finding the genes causally affecting a gene of interest, 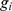, dubbed the *target gene*; in this case, we set 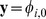, the expression profile of 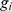 corresponding to time 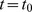. The idea is to relate the linear regression model governing the dynamics of gene expression level of 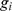 to Equation (1) and use CS techniques to infer 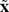; the support of 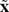 can then be used to identify the genes affecting 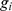. In addition, the sign of the nonzero entries of 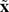 and their values can be used to infer if a gene positively or negatively regulates another gene.

The sensing matrix is formed using the expression profiles of all the genes other than 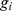, at all past time points, according to the formula:
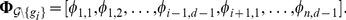
(5)Note that in forming the matrix 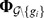, we did not use the expression profiles corresponding to time 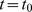. The reason behind this is that we assume that the profile of a gene at time 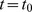 depends on the profile of other genes at *previous* time-points. In addition, we did not include the expression profiles of 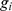 corresponding to past time-points. This is justified by the fact that even if 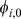 can be written as a linear combination of 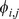, 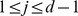, which is the case in most scenarios, it does not imply that the gene self-regulates; as a result, including such columns in the sensing matrix and treating them in the same way as profiles of other genes will result in false-positives and will also mask the effect of the true regulators of the target gene. One should also note that the sensing matrix formed in this way may not satisfy some of the properties reported in CS literature for analytical performance guarantees. For example, there is no guarantee that the matrix will satisfy an RIP-like condition; the RIP is a *sufficient condition* for recovery and may not be necessary for the algorithm to work. Our results, similar to the results pertaining to face recognition [44] show that many interactions can be inferred using this approach despite the fact that the sensing matrices may not satisfy the RIP.

List-SP infers gene interactions based on 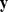 and 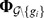, as summarized in Algorithm 1 (Table 1). This algorithm identifies genes that accurately “explain” the behavior of the target gene, which include, but are not restricted to, genes causally influencing the target gene. Hence, the set 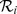 may contain false-positives.

**Table 1:** Algorithm 1: List-SP.

**Input:** 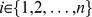 , 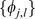 , and 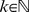
**Output:** 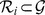
**Initialization:** 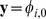 , 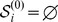 , 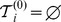 ; form 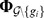
**For** 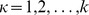 **do**
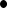 Run SP for vector 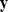 , sensing matrix 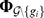 , and sparsity 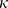
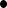 Form 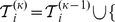 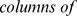 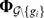 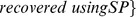
**End**
**Return** 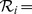 the set of genes corresponding to columns in 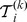

The pseudocode corresponding to the List-SP algorithm.

In order to identify which genes in the set 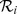 causally influence 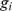, we perform an F-test for each 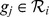, for a given significance level, 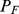. For this purpose, we need to calculate the vector of residuals in the unrestricted regression model and restricted regression model. Let 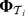 be a matrix formed using the columns in 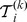 recovered by Algorithm 1. The residual vector of representing 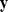 as a linear combination of the columns in 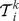 (i.e. the residual vector of unrestricted model), is calculated according to 
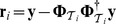
, where “
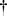
” denotes the Moore-Penrose pseudo-inverse [52]. For each 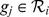, we form 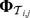 by removing the columns of 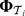 corresponding to 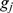. The vector of residuals for each 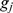 in the restricted model is computed according to 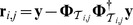. These residual vectors are used in Equation (4), the result of which is subsequently used to reject or accept the hypothesis that 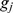 is conditionally Granger-causal for 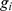, for significance level 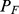. The main steps of the procedure are summarized in Algorithm 2 (Table 2), and the method is termed Causal Subspace Pursuit for Inference and Analysis of Networks (CaSPIAN).

**Table 2:** Algorithm 2: CaSPIAN.

**Input:** 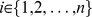 , 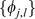 , 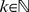 , and 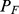
**Output:** 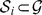
**Initialization:** 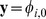 , 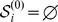 , 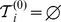 ; form 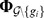
**For** 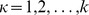 **do**
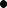 Run SP for vector 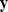 , sensing matrix 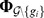 , and sparsity 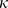
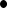 Form 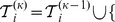 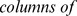 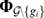 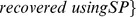
**End**
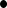 Form 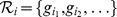
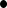 Form 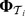 using 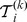 and set 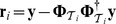
**For** 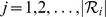 **do**
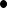 Form 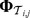 and calculate 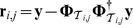
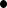 Form the F-statistic using 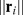 and 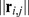
**If** the F-statistic is greater than critical value corresponding to 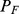
**Then** set 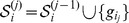
**Else** set 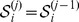
**End**
**Return** 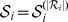

The pseudocode corresponding to the CaSPIAN algorithm.

### CaSPIAN with prior subnetwork knowledge

In many practical situations, some directed edges in the network are known in advance, due to extensive experimental confirmations of their existence. In such cases, one can leverage this side-information to improve the performance of CaSPIAN, especially when the number of time points available for inference is small. For any 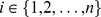, let 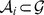 be the set of genes in the known subnetwork that causally influence 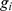. The idea is to first remove the influence of the set of genes in 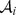 from 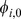, and then to run CaSPIAN on the residual vector. Let 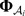 be a matrix formed by setting the profiles of genes in 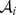 as its columns. Using standard least square methods, the best representation of 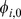 as a linear combination of the columns of 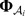 is equal to 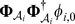, where “
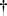
”, as before, denotes the Moore-Penrose pseudo-inverse. As a result, we set 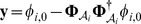, and run CaSPIAN for this choice of 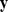 and the sensing matrix formed by all the profiles of genes in 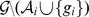. The steps of this method are presented in Algorithm 3 (Table 3).

**Table 3:** Algorithm 3: CaSPIAN given a known subnetwork.

**Input:** 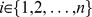 , 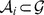 , 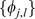 , 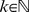 , and 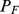
**Output:** 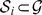
**Initialization:** Form 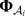 and 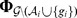 ; set 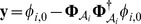 , 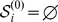 , and 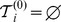
**For** 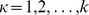 **do**
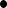 Run SP for vector 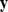 , sensing matrix 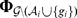 , and sparsity 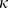
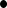 Form 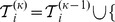 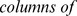 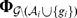 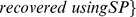
**End**
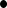 Form 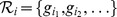
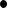 Form 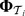 using 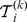 and set 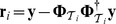
**For** 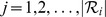 **do**
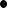 Form 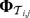 and calculate 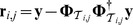
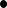 Form the F-statistic using 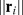 and 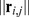
**If** the F-statistic is greater than the critical value corresponding to 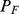
**Then** set 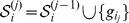
**Else** set 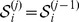
**End**
**Return** 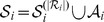

The pseudocode corresponding to the CaSPIAN algorithm given a known subnetwork.

Note that this approach of including the given side-information may not be optimal: one may try to include the information of existing edges into the subspace selection process of the SP method directly, at each of its iteration. Unfortunately, this approach may be computationally more demanding than using pseudo-inversion followed by List-SP and will not be discussed in this paper.

### Forming gene expression profiles

The gene expression profiles 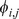, 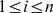 and 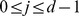, can be formed in different ways. If a large number of experiments (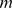) is provided, and each experiment includes exactly 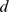 time-points, one can form 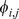 as a vector of length 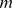 including all the expression levels of gene 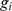 at time 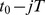. This method was used in [2]. There are two main issues associated with forming 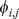 this way. First, 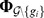 is a matrix of size 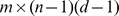; since the number of genes is usually much larger than the number of experiments in a dataset, i.e. 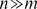, the number of rows in the sensing matrix is much smaller than the number of columns, which significantly deteriorates the performance of any CS-based (or Lasso-based) algorithm. This is due to the fact that the number of columns of 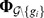 is equal to the number of unknown variables (i.e. length of 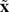), and the number of its rows is equal to the number of known observations (i.e. length of 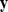). Second, in cases where the number of time-points varies from experiment to experiment, one may not be able to use the additional time-points available in some experiments; the number of “useful” time-points is limited to the minimum number of available time-points over all experiments.

In order to overcome these problems, we use a different method to form 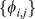. Assume that we want to test if the expression level of a gene 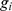 at a given time 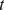 is influenced by the expression levels of other genes at past time-points, considering a time-lag up to 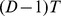, with 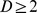. Let 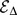 denote the set of experiments for which at least 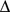 time-points are available, where 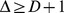. We denote by 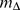 the number of these experiments, i.e. 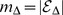. Also, let 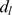, 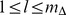, denote the number of time-points available for the 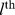 experiment in 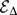. We form 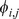, 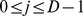 by concatenating the expression levels of 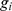 corresponding to a subset of the time-points available in each experiment; the time points we use from the 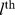 experiment are the expression levels from time 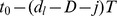 to 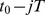, which significantly increases the length of 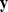 and uses all the available information in the expression data. This results in expression profile vectors of length 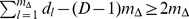. Consequently, the sensing matrix is of size 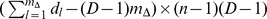.

### Normalization of expression data

Two normalizations are performed on the vectors of gene expressions prior to running the algorithms. Given an expression profile, 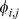, we first subtract the average expression level of this vector from each of its entries. Hence, the normalized profile contains both positive and negative entries. After this step, we normalize the values such that the 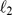 norm of each profile is equal to one. The reason for these normalizations are to reduce the bias and non-uniformities in the expression level of different genes and ensure proper conditions for the operation of the list-SP method. In addition to normalization, we also add an all-one column to the sensing matrix. This step allows us to capture the effect of steady-state values of the profile of the target gene.

### Choosing the parameters of CaSPIAN

CaSPIAN has two input parameters, 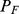 and 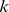. The value of 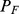 depends on the application at hand. As the parameter 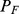 is used as a threshold in the F-test to infer Granger-causality, if 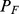 is very small, the number of false-positives is very low. This increases the precision of the algorithm. In return, the sensitivity of the algorithm reduces as well. A well accepted range of values for 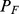 is 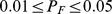 which provides a balance between the sensitivity and precision. On the other hand, in applications where finding true positives is more important than finding all the edges, a significantly smaller value for the parameter may be chosen. We defer an in-depth discussion of this issue to the next section, where we compare results for three different choices of 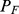 values.

On the other hand, the choice for 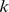 depends on the available information regarding the network and its degree distribution. The compressive sensing SP algorithm [47] assumes that 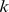 is given a priori. In the context of gene network inference, this is equivalent to knowing the in-degree of each gene in the network. However, by adapting this compressive sensing algorithm to gene network inference applications, the resulting List-SP and CaSPIAN do not require the knowledge of all in-degrees of nodes in the network. Instead, a somewhat tight upper bound suffices, which may be chosen to be the largest reported degree of a hub gene. Hubs in GRNs and protein-protein interactions may have degrees ranging between 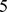 and 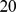, as discussed in [55]. If the value of 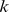 is chosen slightly smaller than the maximum in-degree, the performance of CaSPIAN does not deteriorate noticeably. On the other hand, a highly overestimated value of 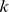 increases the number of false-positives in the list-SP algorithm; however, the F-test embedded in CaSPIAN reduces the number of false-positives. The effect of different choices of 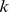 compared to the maximum in-degree of the network is discussed in the next section as well as in the supporting information.

## Results and Discussion

We evaluated the performance of the proposed algorithms with respect to the choice of different parameters such as the sparsity level 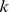, the significance value 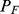, the topology of network, the noise level, and the time-point sampling method. In addition, we compared the performance of these algorithms with that of other causal inference algorithms.

In order to evaluate the effect of different parameters on the performance of the algorithms, we employed synthetic (simulated) networks. Synthetic networks have tightly controlled design parameters, such as the maximum and minimum degree, degree distribution, gene expressions' dynamics, noise level, and sampling frequency. Hence, they allow for accurate assessment of the effect of different parameters on the performance of reverse engineering methods.

On the other hand, synthetic network models usually lack a number of complex features of biological networks that may be hard to model or unknown to the designer. As a result, a fair comparison of different reverse engineering methods require using biological networks. Consequently, we used the IRMA network in *Saccharomyces cerevisiae* [48], the human HeLa cell network, and the SOS network of *E. coli* [49]) to compare CaSPIAN with other known reverse engineering algorithms.

We also used IRMA and human HeLa cell networks to discuss the effect of side-information on the performance of Algorithm 3. In particular, we addressed the effect of employing a known *correct* subnetwork on the performance of CaSPIAN. In addition, we addressed the effect of using an *incorrect* subnetwork on the performance of this algorithm.

In our comparisons, we used four standard evaluation measures, “sensitivity” (recall), “precision”, “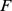-measure” (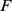-score, not to be confused with the Granger 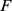 statistics) and “accuracy”. These measures are defined as 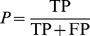, 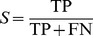, 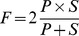 and 
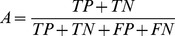
, respectively; “TP” stands for the number of true-positives, “FP” stands for the number of false-positives, “TN” stands for the number of true-negatives, and “FN” stands for the number of false-negatives.

### Influence of different parameters on the performance of the algorithms

As described earlier, we used synthetic networks to evaluate the performance of CaSPIAN with respect to different parameters. The constructed synthetic networks follow a model representing a modification of the Erdös-Rényi model with controlled degree distributions and with additional features that allow its dynamics to converge to a steady-state. A detailed description of the model, which we subsequently refer to as the synthetic network, is given in the supporting information.

#### The over-sampled regime with noise-free expressions

We start by evaluating the performance of SP, List-SP, and CaSPIAN for different values of 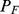, when the number of time-points is larger than the number of genes in the network. We randomly generated 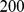 synthetic networks comprising 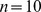 genes that are described with the accompanying performance plots. We selected these parameters given that all the biological networks analyzed in the subsequent sections will have a number of nodes of this order. In addition, given that the number of available time points for analysis of regulatory networks usually does not exceed a dozen, larger networks are unlikely to be inferred with any of the existing methods.

Figures S1 and S5 in Appendix S1 illustrate the average sensitivity of different algorithms with respect to 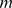 (the length of the gene time-series profiles), for different values of 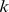 and for two different distributions used for forming the gene expressions (as detailed in the supporting information). We ran CaSPIAN with 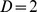 (one time unit lag) and for three significance values: 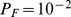, 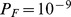, and 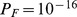. These widely different significance values allow us to evaluate the effect of 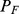 on the performance of the method. As can be seen, the sensitivity of List-SP is higher than SP and CaSPIAN. This is a consequence of the fact that List-SP is specifically designed to increase the TP rate of SP, which consequently increases sensitivity. On the other hand, CaSPIAN uses an F-test to reduce the false-positive rate of List-SP, which may in turn reduce the sensitivity due to an increase in the false-negative rate. The sensitivity of CaSPIAN improves given more time-points (i.e., larger 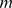), as expected. Another important observation is that the sensitivity of List-SP improves when increasing 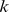. This is due to the iterative structure of List-SP: for any 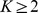, the output of List-SP for 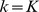 is included in the output of List-SP for 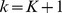. When 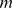 is sufficiently large (roughly 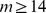) and 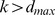, the sensitivity of CaSPIAN is not significantly affected by the choice of 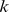. Therefore, one can choose 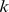 based on a rough estimate of the largest in-degree of the network.

Figures S2 and S6 in Appendix S1 illustrate the average precision of our algorithms with respect to 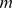. The precision of CaSPIAN is significantly better than the precision of List-SP and SP, even when a relatively large value of 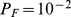 is chosen. The precision of CaSPIAN increases as 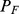 decreases, which is to be expected as a smaller value of 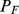 implies a stricter condition for the F-test. However, choosing a very small value for 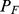 decreases the sensitivity of CaSPIAN. Similar to the case of sensitivity analysis, it may be observed that for sufficiently large values of 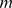 (e.g., 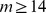) the precision of CaSPIAN is not significantly affected by the choice of 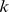.

Figures S3 and S7 in Appendix S1 illustrate the average accuracy, while Figures S4 and S8 in Appendix S1 show the average 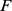-measure of our algorithms with respect to 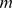. The accuracy and 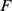-measure of CaSPIAN are significantly better than those of SP and List-SP, and the accuracy and 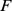-measure of CaSPIAN are not significantly affected by the choice of 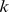 provided that 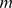 is sufficiently large.

#### The under-sampled regime with noise-free expressions

We also evaluated the performance of the CaSPIAN algorithm when the number of genes is larger than the number of available time-points. Since CaSPIAN is based on a compressive sensing approach, in principle one may use this method when the number of genes is significantly larger than the number of measurements. The impediment of direct use of CaSPIAN on large biological networks is associated with noise in the measurements and the sampling irregularities of experimental data.

We evaluated CaSPIAN and related algorithms on 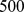 randomly generated networks comprising 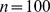 genes, and with expression profiles of size 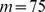, as described in the supporting information. Figures S9 and S11 in Appendix S1 show the average sensitivity, precision, accuracy and 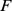-measure of SP, List-SP and CaSPIAN versus the sparsity level 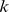, each corresponding to a different degree distribution. The standard deviations corresponding to these measures are provided in separate plots, as shown in Figures S10 and S12 in Appendix S1.

From Figure S9 in Appendix S1, we see that in the absence of noise, List-SP and CaSPIAN outperform SP; in particular, for 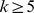, the sensitivity of List-SP and CaSPIAN is at least 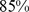, and it grows with 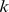. The high sensitivities of these two algorithms imply that at least 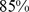 of the directed edges in the network were detected for 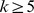. Note that the maximum in-degree of the simulated network was set to 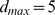. As 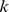 increases beyond 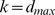, the number of false-negatives decreases, and consequently the sensitivity increases. One can see that both SP and List-SP have low precision when compared to the CaSPIAN algorithm, as the latter applies a Granger-causality test that significantly reduces the number of false-positives. One measure that can capture the joint effects of sensitivity and precision is the 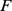-measure, which we analyzed in addition to sensitivity and precision. As for the case of the oversampled regime, a rough estimate of 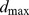 can be used as a bound on 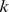 even in the under-sampled regime without significantly affecting the 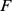-measure of CaSPIAN. Similar results are shown in Figure S11 in Appendix S1. In particular, all values of 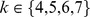 result in nearly identical 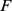-measures for the CaSPIAN algorithm in the under-sampled regime. Note that the networks randomly generated in Figure S11 in Appendix S1 have a maximum in-degree of 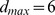.

The results discussed up to this point were based on uniform sampling strategies for the synthetic network, i.e., on datasets obtained by measuring gene expressions at uniformly spaced times. Since in some available datasets measurements were generated using non-uniformly spaced time-points, we examined how the performance of our greedy algorithms is affected by the sampling strategy. For this purpose, 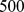 networks of 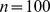 genes were generated randomly as described in the supporting information section. For each network, 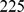 time-points were generated; subsequently, for each network, 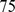 measurements out of the existing 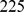 measurements were chosen uniformly at random. This is equivalent to a nonuniform sampling of the original time-points with an average rate of 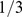. Figures S13 and S14 in Appendix S1 show the effect of nonuniform sampling. As can be seen in these figures, the performance of all the algorithms significantly deteriorates, which suggests that uniform sampling should be practiced in place of nonuniform sampling whenever possible, given that in the model delays are assumed to be regular.

#### The under-sampled and over-sampled regimes with noisy expressions

In order to evaluate the performance of the proposed algorithms in the presence of noise, we considered 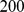 randomly generated networks of 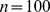 genes. White Gaussian noise with a variance equal to 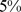 of the average signal power was added to *all* gene expression profiles. The construction of the underlying networks is discussed in Appendix S1.

Figures S15–S22 in Appendix S1 illustrate the mean and standard deviation of the proposed algorithms for different values of 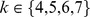 versus the number of time points 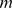; a range of 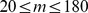 was considered for the number of time points to include both the under-sampled and over-sampled regimes. As can be seen in these figures, List-SP has a higher sensitivity compared to that of the other algorithms; however, CaSPIAN with 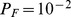 closely follows the sensitivity of List-SP. On the other hand, CaSPIAN with 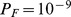 and 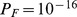 has much smaller sensitivity compared to List-SP.

The opposite behavior can be observed with respect to the precision of these algorithms: the precision of CaSPIAN with 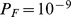 and 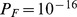 is significantly better than the precision of CaSPIAN with 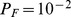, SP, and List-SP. Figures S15–S22 in Appendix S1 show that CaSPIAN has a high accuracy, and the accuracy is not significantly affected by the choice of 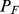. In addition, it can be observed that CaSPIAN with 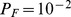 outperforms SP and List-SP with regards to the 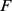-measure in both the under-sampled (i.e. 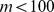) and the over-sampled (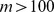) regimes. On the other hand, for 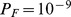 and 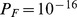, the 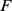-measure increases significantly as 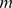 increases.

Next, we focus on the performance of these algorithms in the under-sampled regime in the presence of white Gaussian noise. Figures S23 and S24 in Appendix S1 demonstrate the mean and standard deviation of the sensitivity, precision, accuracy and 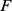-measure of SP, List-SP, and CaSPIAN as a function of the ratio of the noise variance and the average signal power. As expected, the presence of noise deteriorates the performance of all the algorithms; in particular, since CaSPIAN rejects some recovered genes through an F-test, in the presence of noise this may cause some correctly identified edges recovered by List-SP to be rejected. This effect is more prominent for smaller values of 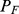. Although the presence of noise increases the number of false-negatives in CaSPIAN, it is important to note that the *precision* of CaSPIAN remains high. In particular, for 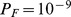 and 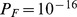, a precision higher than 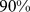 can be maintained for the chosen noise energy.

These findings demonstrate that changing the significance value 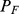 results in a trade-off between sensitivity and precision when noise is present in the system: higher sensitivity may be achieved by choosing a larger value for 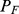 – this is of importance for applications where reducing the false-negative rate is more important than reducing the false-positive rate. On the other hand, by choosing small values for 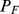, one may be able to detect true-positives with very high reliability, while missing some existing edges; such choices of 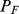 are particularly useful when finding *correct edges* is more important than finding *all edges*. On the other hand, when both sensitivity and precision are equally important, one should use the 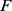-measure to evaluate the performance. In this case, 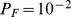 outperforms other choices of 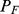, which confirms that a range 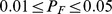 is the most appropriate choice in noisy scenarios, as previously suggested in the literature.

One can also take advantage of the edges recovered using different values of 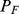 by assigning a confidence level to each edge depending on the 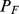 value used to recover that edge. For example, when using the three values chosen for 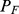 in this section, the edges recovered using 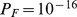 have the highest confidence level; on the other hand, the edges that were recovered for the first time using 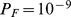 (i.e. they were not present among the edges recovered using 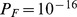) have the second highest confidence level, while the edges recovered for the first time using 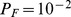 have the lowest confidence level among the others.

### Comparison of CaSPIAN with other reverse-engineering algorithms

As discussed at the beginning of this section, we used in vivo networks to compare the performance of CaSPIAN with other reverse-engineering algorithms. The results are discussed in what follows.

#### The IRMA network in *Saccharomyces cerevisiae*

We focus next on a network model that we believe to be a benchmark standard, given that it shares many features of synthetic networks – such as precise design parameters and controllability – while being part of a biological network in a living organism. The network in question is the IRMA (in vivo reverse-engineering and modeling assessment) network, a synthetic network of five genes, *CBF1*, *GAL4*, *SWI5*, *GAL80*, *ASH1*, embedded in *Saccharomyces cerevisiae* (yeast). The IRMA network has a fixed topology, and is constructed in such a way that its constituent genes are not regulated by other yeast genes. This network was introduced in [48].

**Figure 1:**
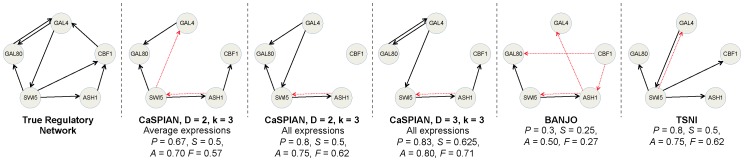
The gene regulatory network corresponding to the IRMA network, obtained using different algorithms. Solid arrows denote true-positives and dashed arrows denote false-positives. True-negatives and false-negatives are not depicted in the figures in order to avoid cluttering; however, they can be easily obtained by comparing the true regulatory network and the inferred networks. Precision is denoted by 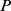, sensitivity by 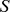, accuracy by 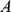, and the 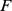-measure by 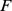.

For our analysis, we used the time-series gene expressions of “switch-on” experiments (shifting cells from glucose to galactose), measured via quantitative real-time PCR (q-PCR) every 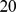 minutes for up to 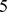 hours, within five experiments. The performance of two additional algorithms utilizing sparsity and causality, TSNI [56] and BANJO [57], were evaluated in [8] using the same dataset. TSNI is an algorithm based on modeling networks via ordinary differential equations (ODE), while BANJO is an algorithm based on Bayesian networks. The reconstructed networks corresponding to these algorithms are depicted in Figure 1.

We first ran CaSPIAN (with 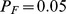, 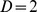, 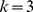) on the time-series dataset, which consisted of the *average* of the gene expressions of the five experiments. As can be seen in Figure 1, CaSPIAN (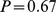, 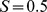, 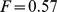) outperforms BANJO (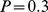, 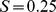, 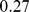); however, its precision and 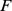-measure are not as good as that of the ODE-based algorithm: TSNI (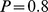, 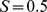, 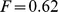). We next ran CaSPIAN using the combination of the gene expressions of the five experiments, employing the method described in the section “Forming gene expression profiles”. As can be seen in Figure 1, CaSPIAN with 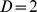 and 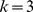 matches the performance of TSNI, while it outperforms TSNI with 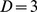 and 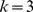 (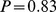, 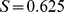, 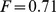). This shows that using data averaged over different experiments causes a significant loss in the available information and deteriorates the performance of CaSPIAN. On the other hand, if one uses all the expressions to form profiles as previously described, the performance of CaSPIAN significantly improves. Note that CaSPIAN outperforms TSNI, in spite of the fact that TSNI uses a cumbersome and complicated procedure including a cubic smoothing spline filter and Principle Component Analysis (PCA) for dimensionality reduction.

As a concluding remark, we point out the interesting fact that the union of edges discovered by CaSPIAN and TSNI includes 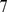 out of 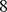 correct edges of the IRMA network and two false positives. This suggests an approach that we believe to be important for future investigation: fusing the outputs of a number of methods tuned to operate under different modeling assumptions. The topic of network fusion is beyond the scope of this paper.

#### A HeLa cell line network

In order to compare the performance of CaSPIAN with that of other algorithms on experimental biological data, we used a network consisting of 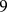 HeLa cell genes. The network corresponding to this set of genes was reported in [58] and was used in the literature as a benchmark for evaluating the performance of different algorithms such as CNET [58], Group Lasso (grpLasso) [59] and truncating adaptive Lasso (TAlasso) [2]. CNET is an algorithm for reverse engineering of causal gene interactions using microarray time series data; this algorithm tries to find the “best” directed graph among all the possible graphs, where the quality of a candidate graph is determined using a specific scoring function. On the other hand, grpLasso and TAlasso are penalty-based algorithms that rely on 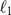 minimization. The grpLasso algorithm infers causal interactions by evaluating the effect of different gene expressions *averaged* over different time-lags and therefore does not take advantage of all the information available in the time-series data. On the other hand, TAlasso does not suffer from the loss of information in grpLasso and employs an additional truncation method to simplify the model. However, as discussed in earlier sections, it suffers from the shortcomings intrinsic to 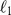 minimization based Lasso approaches.

**Figure 2:**
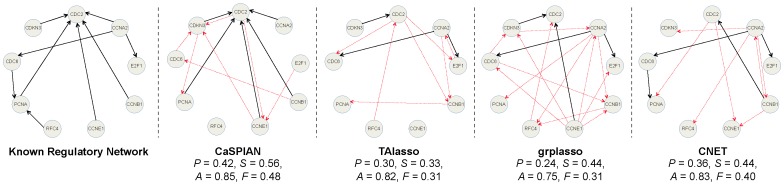
A gene regulatory network of HeLa cell genes, reconstructed using different causal inference algorithms. Solid arrows denote true-positives and dashed arrows denote false-positives. True-negatives and false-negatives are not depicted in the figures to avoid cluttering; however, they can be easily obtained by comparing the known regulatory network and the inferred network. Precision is denoted by 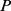, sensitivity by 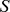, accuracy by 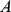 and the 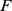-measure by 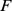.

We used the expression data of HeLa cells from the third experiment performed in [60], consisting of 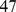 time-points. For each algorithm, we used the reconstructed graphs reported in the corresponding papers (as pointed out in Footnote 1 of [2], there appear to be some errors in the reported results of [59]; therefore, we used the corrected results of [2]). Since in [2] three time lags were used for TAlasso, we also used three time-lags in CaSPIAN (or equivalently, we set 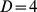). Since the true in-degree of the network is 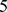, we used 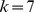 as a rough upper bound. In addition, we set 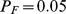 as a widely accepted significance value for 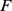-tests. The results are shown in Figure 2, along with the precision, sensitivity and accuracy for each algorithm.

As can be seen in this figure, CaSPIAN outperforms all the other algorithms with respect to all three parameters: precision, sensitivity and 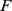-measure. Note that this is in spite of the fact that CNET is a search-based algorithm and performs an extensive search to find the best graph. As a result, not only does CaSPIAN provide higher efficiency than the other algorithms, but it also outperforms them in terms of recovering the gene regulatory network. It is again interesting to point out that CaSPIAN and CNET tend to discover almost disjoint sets of true-positives, which suggests combining the methods as described for the IRMA network.

#### The SOS genes in *E. coli*

Our analysis of the IRMA and HeLA networks illustrated the performance of CaSPIAN and other causal inference algorithms for the case when the number of time-points was larger than the number of genes (corresponding to the over-sampled regime). As CaSPIAN is based on compressive sensing methods which allow for inference in the under-sampled regime, and as most inference problems operate in the under-sampled regime, it is instructive to test the performance of the method on a larger network. The performance of CaSPIAN is contrasted to that of the Lasso and truncating Lasso (Tlasso) [2].

We used the gene expression data of *E. coli* from the Many Microbe Microarrays Database (M3D) [61]. To evaluate the performance of CaSPIAN, we focused on the SOS subnetwork. The main documented genes in this subnetwork are *dinI*, *lexA*, *recA*, *recF*, *rpoD*, *rpoH*, *rpoS*, *ssb*, *umuC/D*, as described in [49]. We denote these genes by 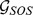. The known links in the SOS network of *E. coli* are available in Table S4 of [22]; for completeness, and as a reference for our comparisons, we provided this information in Appendix S2. The M3D database contained the expression levels of 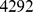 genes from 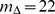 experiments with at least 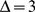 time-points. The number of time-points in these 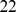 experiments was at least 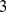 and at most 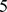. Using the method for forming gene expression lists outlined in the previous section, we combined the data of the 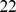 experiments producing profiles of length 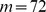. In order to evaluate the performance of these algorithms in the *under-sampled* regime, we randomly selected a set of 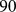 genes from the 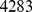 genes other than 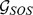 and formed a set of 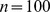 genes as the union of this set and 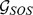. We repeated this procedure 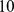 times: we ran List-SP, CaSPIAN, Lasso, and TLasso on 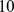 sets, each containing 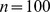 genes, formed as the union of 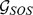 and a set of 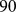 other genes chosen independently and uniformly at random. Executing each algorithm more than once was motivated by the idea of introducing the multiplicity ratio (MR) as a measure of confidence for the recovered links. MR represents the number of runs of the algorithm that produced a given correct edge, divided by the total number of runs of the algorithm.

**Figure 3:**
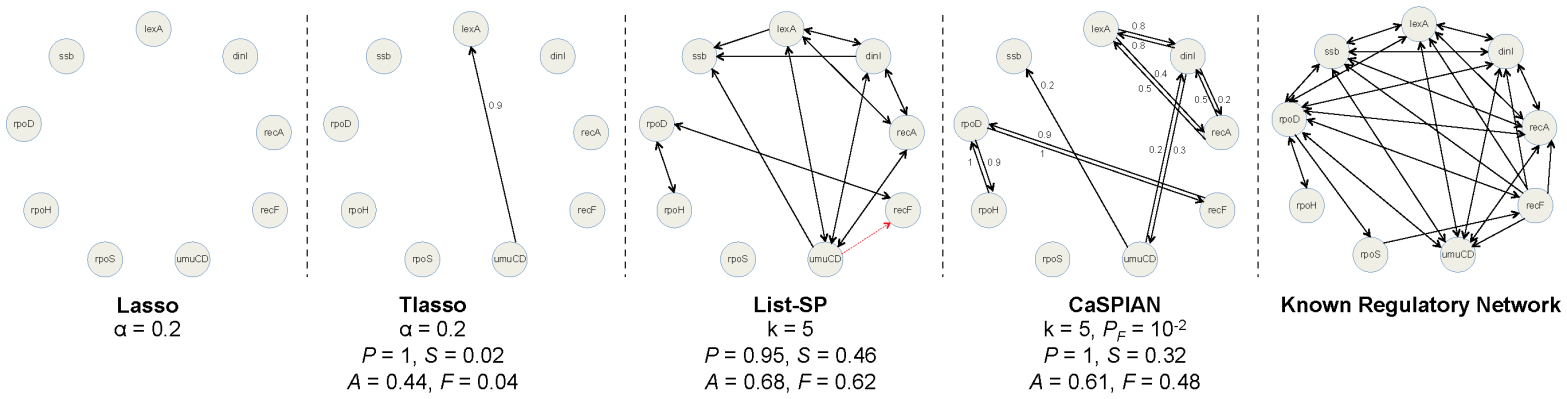
The SOS network reconstructed using Lasso, TLasso, Algorithm 1 (List-SP) and CaSPIAN. Black solid arrows correspond to true positives and red dashed arrows correspond to false-positives. The numbers above edges describe their multiplicity ratios (MRs); in order to avoid cluttering, we did not plot the MRs for the results of Algorithm 1. Note that only links with MR at least 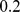 are shown. Precision is denoted by 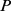, sensitivity by 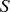, accuracy by 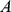 and the 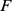-measure by 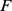.

We ran List-SP (Algorithm 1) for 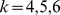 using the gene expression profiles formed according to the method of the previous section, and with 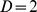. Appendix S2 provides the tables of MRs for the recovered directed edges corresponding to 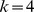, 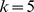, and 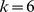. Figure 3 shows the network reconstructed using List-SP (with 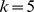 and MR at least 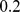). Comparing this figure with the results in [49] reveals that List-SP is capable of correctly identifying 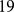 edges in the SOS network (note that there exists a number of feedback loops between two vertices that are counted as two separate edges). However, List-SP misses some edges in the network due to the choice of 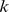 and noise in the expression data. Using a larger value of 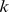 for List-SP allows us to find more existing links in the network, but the number of false-positives increases as well, which is expected (see tables in Appendix S2). We ran CaSPIAN for 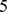 different values of 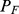 on the generated 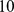 sets of genes. The corresponding MR values of the links found using CaSPIAN are shown in tables in Appendix S2. The reconstructed network for 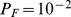 is shown in Figure 3. It is important to note that *all* the links found using CaSPIAN are correct links (i.e., the regulatory relationships have been reported in the literature).

We ran Lasso and Tlasso on the same set of genes, both of which are combinations of Granger causality with different versions of a Lasso penalty (the codes for these algorithms are available at http://www.biostat.washington.edu/~ashojaie). We used the *error based method* for choosing the regularization coefficient. In [2], the parameter 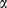 was chosen between 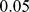 and 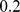, and it was stated that different values of 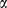 do not affect the performance of the algorithm significantly. As a result, we picked 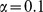 (the default value in the algorithm) and 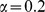. For a detailed description of the error based method and the definition of 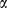, see the original paper [2]. Default values were used for all other parameters in these two algorithms. Lasso was unable to find even a single correct link between the SOS genes; similarly, Tlasso failed to find any links among the genes for 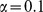. On the other hand, for 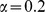, it correctly identified a link from *umuC/D* to *lexA* with MR equal to 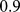; however, no other link was found using this method. Comparing these results with List-SP and CaSPIAN reveals that both of these algorithms outperform Lasso and Tlasso in the undersampled regime. As another illustrative example, CaSPIAN with 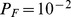 found 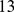 true positives and 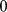 false-positives, while Tlasso only found one true positive.

### CaSPIAN with scaffolding subnetworks

We examined the performance of CaSPIAN assuming that some directed edges in the network are known in advance (Algorithm 3). In particular, we evaluated the improvement/change in the performance of CaSPIAN given that a *correct* subnetwork is known. In addition, we assessed the degradation/change in the performance of CaSPIAN given that an *incorrect* subnetwork is assumed. Since the exact regulatory network of IRMA is known, in our study we mainly focus on the IRMA network.

#### Application to the IRMA network

**Figure 4:**
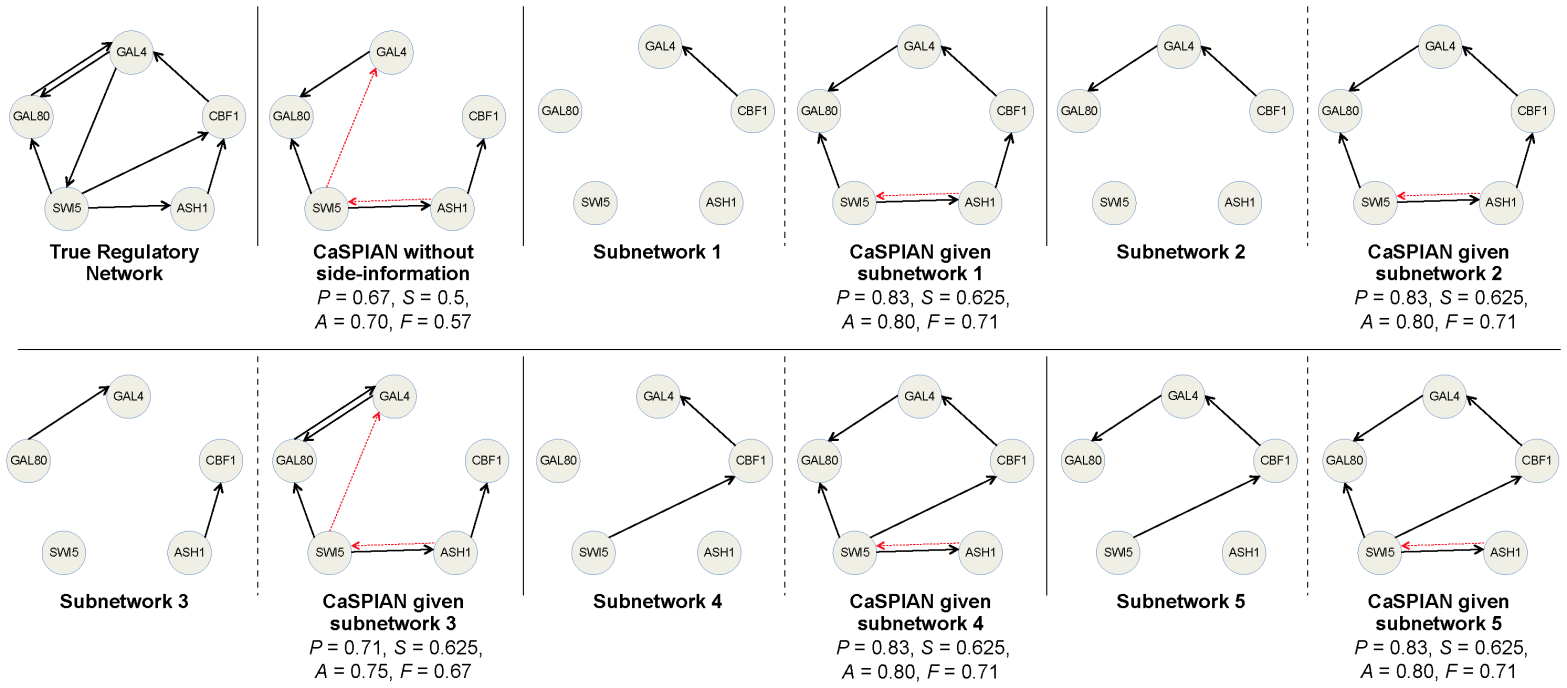
The network corresponding to IRMA obtained by applying Algorithm 3 to various subnetworks. Parameters of this algorithm were chosen as 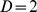 and 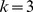 and average gene expressions were used. Solid arrows denote true-positives and dashed arrows denote false-positives. Precision is denoted by 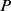, sensitivity by 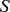, accuracy by 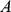, and the 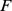-measure by 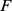.

In order to evaluate the performance of Algorithm 3, we used the averaged expressions of the genes in the IRMA network corresponding to the “switch-on” experiments. We applied Algorithm 3 with parameters 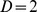 and 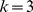 to different known subnetworks consisting of 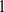, 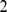, and 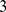 correct edges. Note that we used averaged data and suboptimal CaSPIAN parameters, as the optimal performance of CaSPIAN may not be further improved for the IRMA network given prior information. The results are illustrated in Figure 4. As can be seen in this figure, knowing even one correct edge can improve the performance of CaSPIAN significantly, although in these and most other tests we performed, the number of false positives remained unchanged. As an illustrative example, CaSPIAN without side-information recovers a false-positive edge from *SWI5* to *GAL4*. However, if the information that an edge exists from *CBF1* to *GAL4* is provided a priori, CaSPIAN correctly concludes that no edge exists from *SWI5* to *GAL4*.

**Figure 5:**
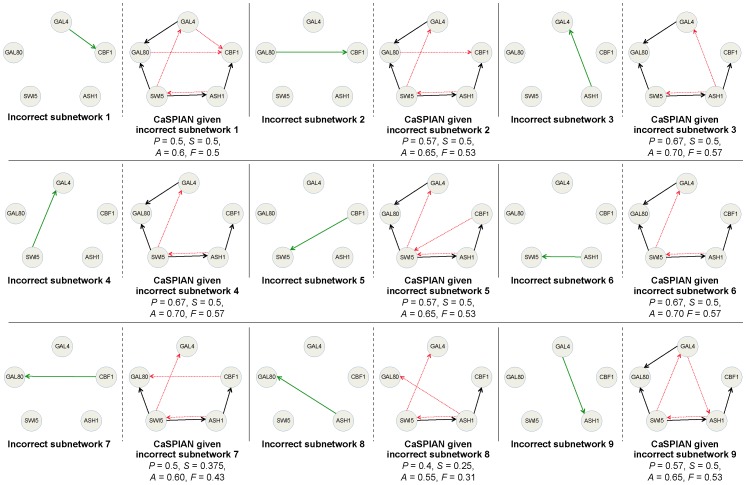
The network corresponding to IRMA obtained by applying Algorithm 3 to various incorrect subnetworks. Parameters of this algorithm were chosen as 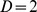 and 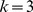 and average gene expressions were used. Solid arrows denote true-positives and dashed arrows denote false-positives. Green solid arrows correspond to the incorrect edges in the subnetwork. Precision is denoted by 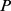, sensitivity is denoted by 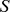, accuracy by 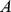, and the 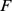-measure by 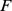.

In order to address the second question, we considered 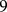 different incorrect edges as a given subnetwork, and applied Algorithm 3 (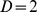 and 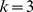) to averaged IRMA expressions. As before, we used suboptimal performance parameters in order to be able to compare the drawbacks/advantages of using incorrect/correct side information, as compared to using no side information, given one and the same dataset. The results are shown in Figure 5. When an incorrect subnetwork is provided, Algorithm 3 forces CaSPIAN to build a network around this structure. Still, when the wrong side-information represents an edge outside the true network, most correctly identified edges remain accurate, but a large number of new false-positives arise.

#### Application to HeLa cell genes

Since the exact regulatory network of *HeLa* is not completely known, we only address the first question raised in the introduction of the section.

**Figure 6:**
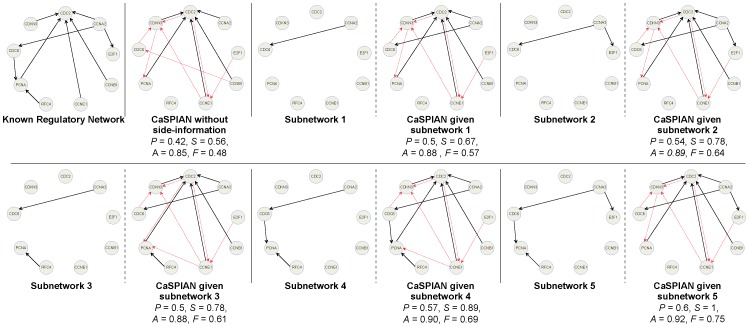
The HeLA network inferred by CaSPIAN with D = 4 and k = 7. Solid arrows denote true-positives and dashed arrows denote false-positives. Precision is denoted by 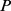, sensitivity is denoted by 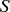, accuracy by 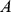, and the 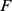-measure by 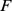.

Figure 6 shows the results of Algorithm 3 applied to different known subnetworks consisting of 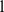, 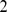, 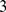, and 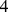 edges. As can be seen, knowing a subnetwork consisting of 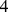 edges suffices to achieve sensitivity equal to 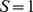. Also, it is important to note that the number of false-positive edges corresponding to the genes claimed to influence *CDKN3* cannot be reduced, since the in-degree of this gene in the known regulatory network is 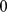. Therefore, the edges found using CaSPIAN for this target gene do not change given the different subnetworks.

## Conclusions

The CaSPIAN approach for network inference represents a new attempt to connect the fields of compressive sensing, causal inference and bioinformatics. Compressive sensing (CS) is a technique for sampling and recovering sparse signals using optimization and/or greedy approaches. In spite of its widespread applications in different areas such as signal processing, image and video processing, communications, etc., its utility has not yet been fully explored in the field of bioinformatics and gene network inference. Although [42] and [43] employ the CS framework to infer gene networks, there are crucial differences between CaSPIAN and the algorithms discussed in these papers. First, unlike CaSPIAN, none of these algorithms are capable of inferring causal (i.e. directed) edges in the gene network. Second, CaSPIAN is an unsupervised learning algorithm that uses CS as the core of its approach; however, [43] introduced an approach which employs CS only as a preprocessing step and combines the obtained results with extensive prior biological information to infer gene networks using a supervised learning algorithm performed by Adaboost. However, due to the susceptibility of Adaboost to random classification noise [45], application of such booster algorithms in biological network inference is limited. Finally, CaSPIAN employs a low-complexity greedy CS approach, which significantly reduces its computational complexity compared to the 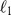 minimization approach used in [43].

Another line of work explored in the literature integrates the sparsity of gene networks using some version of the LASSO penalty [2], [36]–[38]. There are two main difficulties associated with the LASSO approach. First, such methods require performing high dimensional 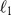 optimization, which has a high computational complexity compared to greedy CS-based algorithms such as CaSPIAN. In addition, in order to enforce the sparsity criterion, these approaches require a proper choice for the coefficient of the regularization term. Unlike CaSPIAN, in which the parameter 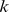 is directly related to the sparsity of the network (i.e. the in degree of the nodes), the regularization coefficient in the LASSO penalty does not directly correspond to the sparsity of the network and therefore is usually chosen using some heuristics or by performing optimization, which increase the complexity of these algorithms without providing provable performance guarantees. On the other hand, by using CaSPIAN, one is able to directly employ information about the degree of the nodes in the network; as we showed in the results, if such information is not available, in most cases an upper bound on the in-degree is sufficient.

CaSPIAN is a reverse engineering algorithm that is based on the list-SP algorithm: a low-complexity greedy search method, which scans the expression profiles for low-dimensional subspaces. Although the computational advantage of the method may not be of great relevance for current small network inference problems, with the ever-increasing volumes of data, this issue will become important in the near future. Furthermore, CaSPIAN does not require fine parameter tuning or supervised learning methods as was shown using both synthetic and real biological data. In addition, in Algorithm 3 we provided a version of CaSPIAN that incorporates biological side-information in the form of scaffolding subnetworks of accurate edges. Our analysis shows that correct edges may significantly improve the performance of CaSPIAN. Given that wrong side-information may severely deteriorate the performance of the inference methods, it may be advisable to use prior information with caution - for example, only if the links were experimentally verified using different techniques by at least several different research labs.

We also evaluated the influence of different parameters such as network topology, degree distribution, size, number of measurements, sampling method, noise, and the value of 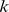 and 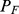 on the performance of the proposed algorithms using synthetic (simulated) networks. In particular, we showed that there exists a tradeoff between the sensitivity and precision of CaSPIAN that is mainly controlled by the choice of 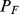. As a result, in applications in which finding a few highly reliable edges is of main interest, one can choose the value of 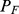 to be small; however, in applications where both precision and sensitivity are of interest, a value of 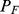 between 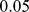 and 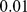 is more appropriate. Another approach to take advantage of this tradeoff is to assign a reliability score to each edge based on the smallest value of 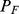 for which CaSPIAN recovered that edge.

In addition, we compared the performance of CaSPIAN with that of other algorithms using real biological (in vivo) networks and showed that in many cases CaSPIAN outperforms other algorithms in spite of their extensive use of resources and side-information and their high complexity. Although we provided extensive comparisons with other models and illustrated that under almost all performance criteria used by the reverse engineering community, CaSPIAN outperforms these methods, we cannot argue that there exists one “optimal approach” for all problems. As an example, we did not test the performance criteria used in the DREAM2 challenge, where teams were asked to provide rank-ordered list of edges believed to exist in the network, according to their reliability. Those lists were truncated to top-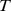 candidates, for different values of 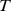, and tested for accuracy and precision. In a different approach, precision and accuracy were calculated at the point of the 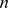-th correct prediction. Each performance criteria is bound to give slightly, if not vastly different answers. It would therefore be of interest to investigate optimal fusion strategies of different methods based on different evaluation criteria in order to recognize the strengths and weaknesses of current methods and fully utilize them in the reverse engineering process. For example, combining CaSPIAN with reverse engineering algorithms based on a completely different framework, such as Bayesian networks or differential equations, may result in a method that can outperform each individual algorithm in a noisy, non uniformly under-sampled regime.

### Availability

The implementation of these algorithms in Matlab will be offered upon request. Please contact the following email address: emad2@illinois.edu.

## Supporting Information

Appendix S1 **CaSPIAN applied to synthetic networks.**
(PDF)

Appendix S2 **Tables of multiplicity ratios (MRs) for the** ***E. coli*** **SOS network.**
(PDF)
