# Supplementary material for: CaSPIAN: A Causal Compressive Sensing Algorithm for Discovering Directed Interactions in Gene Networks
Source: PLoS One. 2014 Mar 12;9(3):e90781. doi: 10.1371/journal.pone.0090781 (PMC3951243; doi:10.1371/journal.pone.0090781)
Supplement: Appendix S1 — CaSPIAN applied to synthetic networks. (PDF) [file pone.0090781.s001.pdf]

Appendix S1: CaSPIAN applied to synthetic networks

Synthetic networks

We used synthetic models to evaluate the effects of different network characteristics, different noise levels, different sampling methods, and different choices for the parameters k an P_F on the performance of our inference approach.

In what follows, we demonstrate how a standard compressed sensing algorithm, SP, performs compared to List-SP and CaSPIAN. Second, we evaluate the effect of different choices of k and P_F on the performance of CaSPIAN. For this purpose, we pick three different values of P_F, i.e. $P_F = 10^{-2}$, $P_F = 10^{-9}$, and $P_F = 10^{-16}$, to cover the largest practical span of values for the rejection probability. Third, we address the effect of noise and non-uniform sampling of gene expressions on the performance of the aforementioned algorithms.

We start by describing our synthetic network model.

Generating the synthetic data

The synthetic networks were constructed in a random fashion, using structural constraints known to exist in biological networks. A number of different random network models have been suggested in the literature, including the Erdös-Rényi $G(n,p)$ model [1], the Erdös-Rényi $G(n,M)$ model [1], directed scale free networks [2], the Albert-Barabási's model [3], and Watts-Strogatz networks [4]. The aforementioned models result in networks with different degree distributions, unless controlled by additional modeling parameters. Since SP, list-SP, and CaSPIAN, all take an estimate of the largest in-degree of the network as an input (i.e., k), in order to evaluate the effect of k, we devised our own random network model in which one can easily control the fraction of nodes with a prescribed in-degree.

For a fixed degree distribution, each network was chosen uniformly and independently at random from all the possible directed graphs with the given in-degree distribution. Note that this simple model may be viewed as a modification of the Erdös-Rényi $G(n,M)$ model, where the degree distribution of the network remains fixed. The choices of degree distributions are described in more details in what follows.

Let $\mathbf{A} \in \mathbb{R}^{n\times n}$ be a matrix such that $\mathbf{A}(i,j) = 0$ if there exists no edge from vertex j to vertex i, and non-zero otherwise. In other words, let $\mathbf{A}$ be the adjacency matrix of the network at hand. Let $\mathbf{g}^{\{l\}} \in \mathbb{R}^n$, $0 \le l \le L$, be a vector with i^{th} entry equal to the expression level of gene i at time $t = t_0 + lT$, and let L be the number of time points. We considered the following model for gene expressions at time $t = t_0 + lT$

$$\mathbf{g}^{\{l\}} = \mathbf{A}\mathbf{g}^{\{l-1\}}, \qquad (1)$$

where $\mathbf{g}^{\{0\}}$ denote the initial values, and $0 \le l \le L$.

Note that we assumed a *linear* model for the dynamics of gene expressions although it is unlikely that in reality gene regulatory networks obey such models. However, given that there is no comprehensive knowledge about the exact dynamics of networks, and given that these dynamics vary from subnetwork to subnetwork, we opted for the simplest modeling scenario. A linear model is also the *most favorable* model for our analysis, since our causal inference algorithms are based on the premise that the dynamics of the network approximately follows a linear model. Nevertheless, we also evaluated the performance of the proposed algorithms on *potentially non-linear* biological networks such as the IRMA network, HeLA cell network, and the SOS network of *E. coli*, as discussed in the paper.

CaSPIAN applied to noise-free gene expressions

The over-sampled regime

First, we evaluate the performance of SP, List-SP, and CaSPIAN for different values of P_F, when the number of time-points L is larger than the number of genes in the network, i.e. $L \geq n$. For this purpose, we used the model described above to randomly generate networks comprising $n = 10$ genes; each network contains 2 genes with in-degree 1, 4 genes with in-degree 2, and 4 genes with in-degree 3. As a result, the number of edges in each network is fixed and equals 22; the topology of the networks are different due to the random nature of the construction process. Note that the degree distribution was chosen in a way to mimic the degree-distribution of known biological networks, such as the *E. coli* SOS network.

First, we tested the aforementioned algorithms on 200 randomly generated networks for which the values of non-zero entries of $\mathbf{A}$ were chosen in an independent identically distributed (i.i.d.) manner, following a normal distribution $\mathcal{N}(0, 25)$. Similarly, the entries of $\mathbf{g}^{\{0\}}$ were chosen in an i.i.d. manner, following a normal distribution $\mathcal{N}(5, 25)$. In order to ensure that the gene expression profiles do not diverge, we normalized the entries of $\mathbf{A}$ by the spectral radius of $\mathbf{A}$, defined as

$$\rho(\mathbf{A}) = \max_i(|\lambda_i|),$$

where λ_i is the ith eigenvalue of $\mathbf{A}$. In order to ensure non-diverging expression profiles, one has to design the update matrix so that the sequence of consecutive powers of $\mathbf{A}$ is convergent. This can be achieved if $\rho(\mathbf{A}) \leq 1$. The performance of these algorithms are shown in Figures S1-S4.

Next, we tested these algorithms on 200 randomly generated networks for which the value of each non-zero entry of $\mathbf{A}$ was chosen in an i.i.d. manner, following a uniform distribution $\mathcal{U}(-5, 5)$. The entries of $\mathbf{g}^{\{0\}}$ were chosen in an i.i.d. manner, following a uniform distribution $\mathcal{U}(0, 20)$. Similar to the previous case, we normalized the entries of $\mathbf{A}$ by the spectral radius of $\mathbf{A}$. The results are shown in Figures S5-S8.

The under-sampled regime

In order to evaluate the performance of the proposed algorithms in the under-sampled regime, we considered networks of $n = 100$ genes, where the fraction of nodes having in-degree l, $d_{min} \leq l \leq d_{max}$ is proportional to $cl^{-\gamma}$. Here, d_{min} and d_{max} denote the minimum and maximum in-degree of the networks, γ is a parameter that determines the rate of decay of the degree distribution, and c is a normalization constant chosen so that $\sum_{l=d_{min}}^{d_{max}} c\, l^{-\gamma} = 1$.

The gene expressions were formed using a matrix $\mathbf{A}$ with non-zero entries following an i.i.d. uniform distribution $\mathcal{U}(-5, 5)$. In order to ensure that the gene expression profiles do not diverge, we normalized the entries of $\mathbf{A}$ by the spectral radius of $\mathbf{A}$. The entries of $\mathbf{g}^{\{0\}}$ where chosen following an i.i.d. uniform distribution $\mathcal{U}(0, 20)$. Note that the uniform distribution corresponds to the least informative prior. We assumed that only $m = L = 75$ time-points are available for the gene expressions.

We considered two sets of parameters. Figures S9 and S10 were obtained by randomly generating 500 networks of $n = 100$ genes with parameters $\gamma = 2.5$, $d_{min} = 2$, and $d_{\max} = 5$. Note that these parameters result in 61 nodes with in-degree 2, 23 nodes with in-degree 3, 11 nodes with in-degree 4, and 5 nodes with in-degree 5, with a total of 260 directed edges.

Figures S11 and S12 were obtained by randomly generating 500 networks of $n = 100$ genes with parameters $\gamma = 2$, $d_{min} = 3$, and $d_{\max} = 6$. Note that these parameters result in 47 nodes with in-degree 3, 26 nodes with in-degree 4, 17 nodes with in-degree 5, and 10 nodes with in-degree 6, with a total of 390 directed edges.

Non-uniform sampling

For the non-uniform sampling case, we generated 500 random networks comprising $n = 100$ genes and parameters $\gamma = 2.5$, $d_{min} = 2$, and $d_{\max} = 5$. For each network, we generated the expression levels for 225 time-points and randomly selected and sorted 75 of the expression values. This results in an average sampling rate of 1/3. Figure S13 and S14 show the effect of non-uniform sampling on the performance of SP, List-SP and CaSPIAN.

CaSPIAN applied to noisy gene expressions

In order to evaluate the performance of the proposed algorithms in the presence of noise, we considered networks of $n = 100$ genes, where the fraction of nodes having in-degree l, $d_{min} \leq l \leq d_{max}$ is proportional to $c\, l^{-\gamma}$. Here, d_{min} and d_{max} denote the minimum and maximum in-degree of the networks, γ is a parameter that determines the rate of decay of the degree distribution, while c is a normalization constant chosen so that $\sum_{l=d_{min}}^{d_{max}} c l^{-\gamma} = 1$. For our evaluations, we chose parameters $d_{min} = 2$, $d_{\max} = 5$, and $\gamma = 2.5$, $d_{\min} = 2$, which result in 61 nodes with in-degree 2, 23 nodes with in-degree 3, 11 nodes with in-degree 4, and 5 nodes with in-degree 5, with a total of 260 directed edges.

The gene expressions were formed using a matrix $\mathbf{A}$ with non-zero entries following an i.i.d. uniform distribution $\mathcal{U}(-5, 5)$. In order to ensure that the gene expression profiles do not diverge, we normalized the entries of $\mathbf{A}$ by the spectral radius of $\mathbf{A}$. The entries of $\mathbf{g}^{\{0\}}$ where chosen in an i.i.d. manner, following a uniform distribution $\mathcal{U}(0, 20)$, which corresponds to the least informative prior. A Gaussian noise was added to the gene expressions.

Figures S15-S22 illustrate the performance measures for different values of k versus the number of time points m using 200 randomly generated networks; a range of $20 \leq m \leq 180$ was considered for the number of time points to include both the under-sampled and over-sampled regimes. A Gaussian noise with a variance equal to 5% of the signal power was used to obtain these results.

In order to evaluate the performance of the proposed algorithms in the under-sampled regime with different noise variances, we fixed $k = 5$ and generated 500 random networks with $n = 100$ genes and parameters $\gamma = 2.5$, $d_{\min} = 2$, and $d_{\max} = 5$. The results are shown in Figures S23 and S24.

References

1. Erdös P, Rényi A (1959) On random graphs. Publicationes Mathematicae 6: 290-297.

2. Bollobás B, Borgs C, Chayes C, Riordan O (2003) Directed scale-free graphs. Proceedings of the 14th ACM-SIAM Symposium on Discrete Algorithms : 132-139.

3. Albert R, Barabási AL (2000) Topology of evolving networks: local events and universality. Phys Rev Lett 85: 5234-5237.

4. Watts DJ, Strogatz SH (1998) Collective dynamics of 'small-world' networks 393: 440-442.

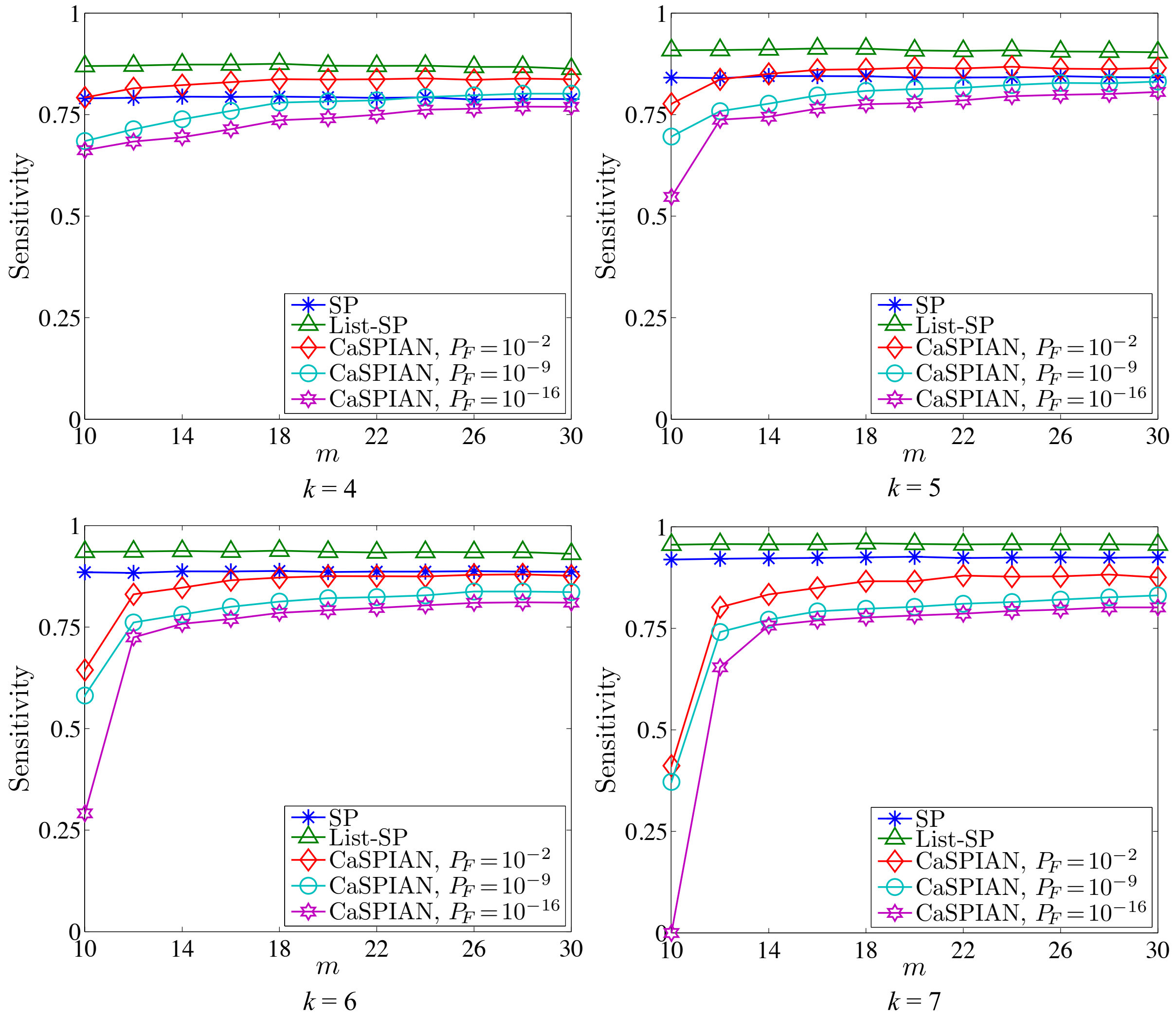

Figure S1. The average sensitivity of different algorithms for Gaussian gene expression model. The average sensitivity of different algorithms as a function of m, obtained from 200 randomly generated networks with $n = 10$; Gaussian distribution was used to form the gene expressions.

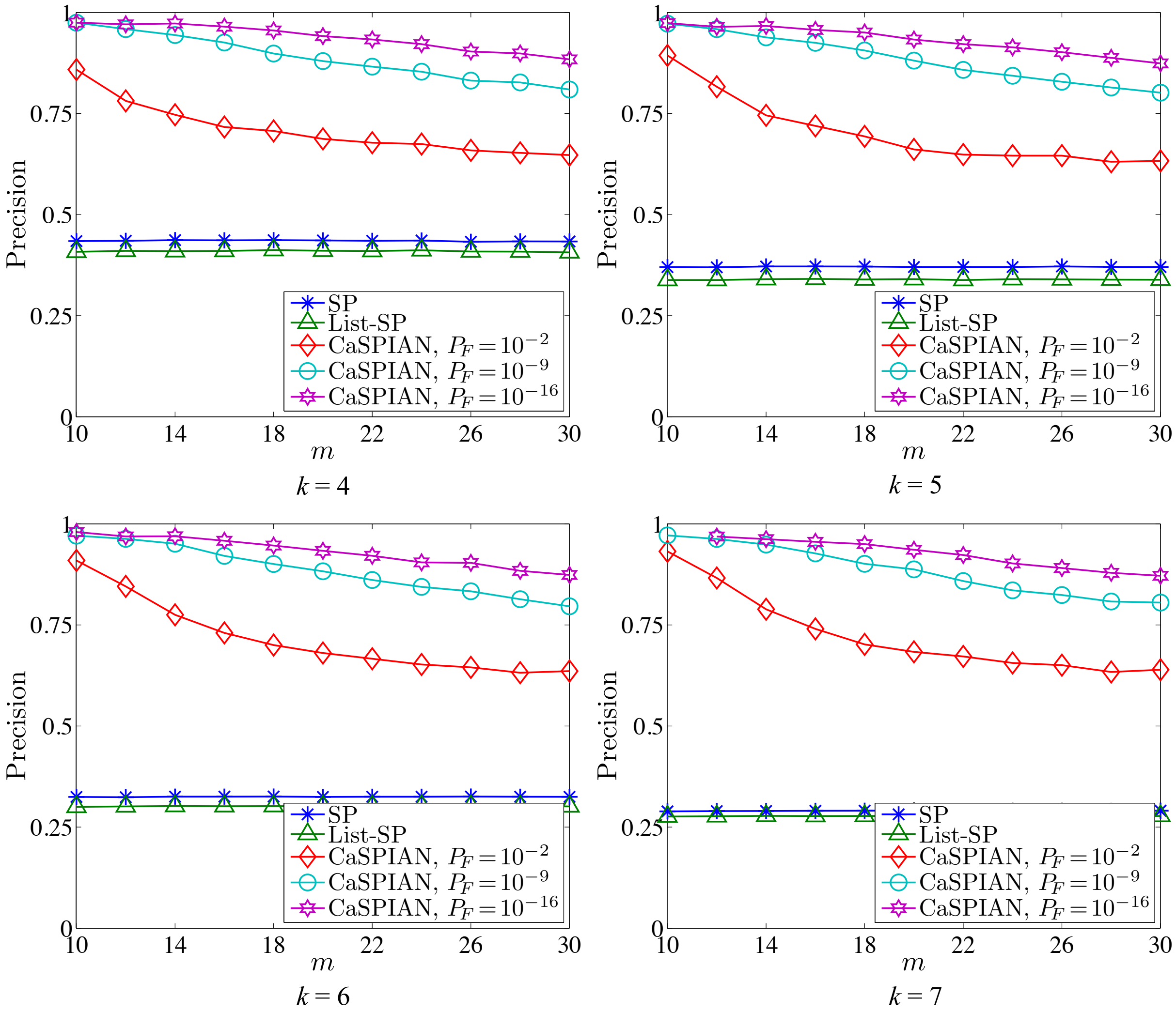

Figure S2. The average precision of different algorithms for Gaussian gene expression model. The average precision of different algorithms as a function of m, obtained from 200 randomly generated networks with $n = 10$; Gaussian distribution was used to form the gene expressions.

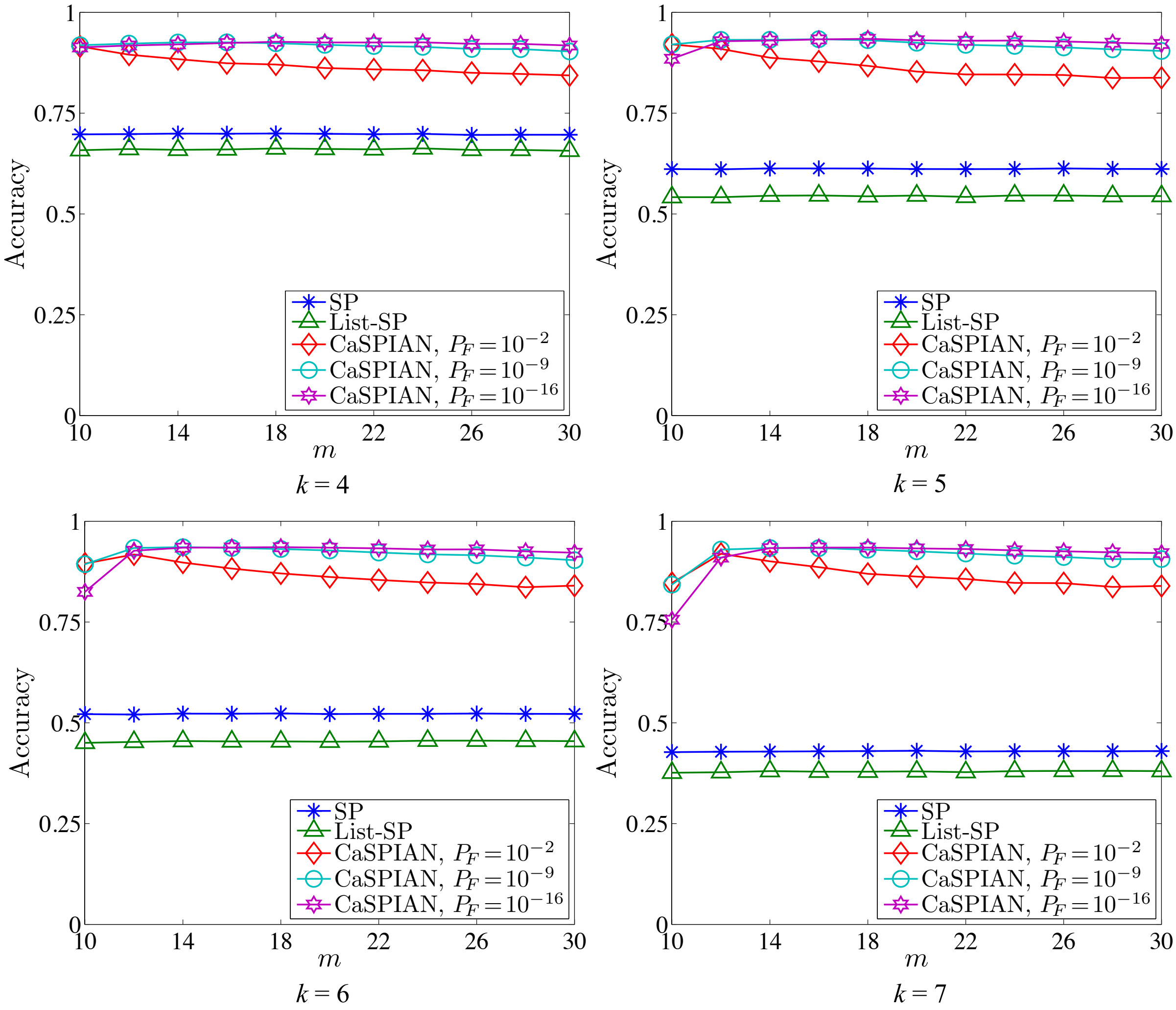

Figure S3. The average accuracy of different algorithms for Gaussian gene expression model. The average accuracy of different algorithms as a function of m, obtained from 200 randomly generated networks with $n = 10$; Gaussian distribution was used to form the gene expressions.

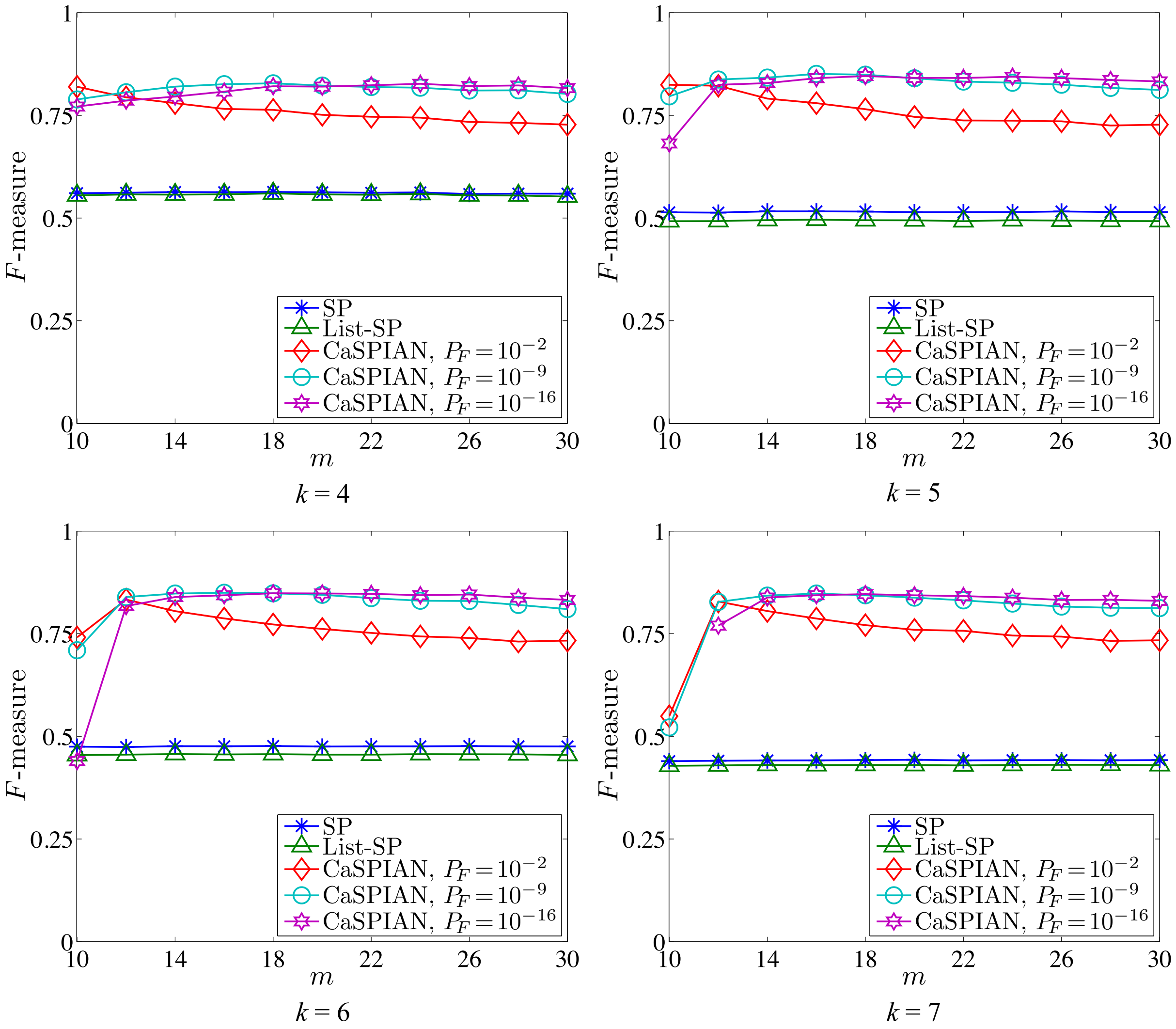

Figure S4. The average F-measure of different algorithms for Gaussian gene expression model. The average F-measure of different algorithms as a function of m, obtained from 200 randomly generated networks with $n = 10$; Gaussian distribution was used to form the gene expressions.

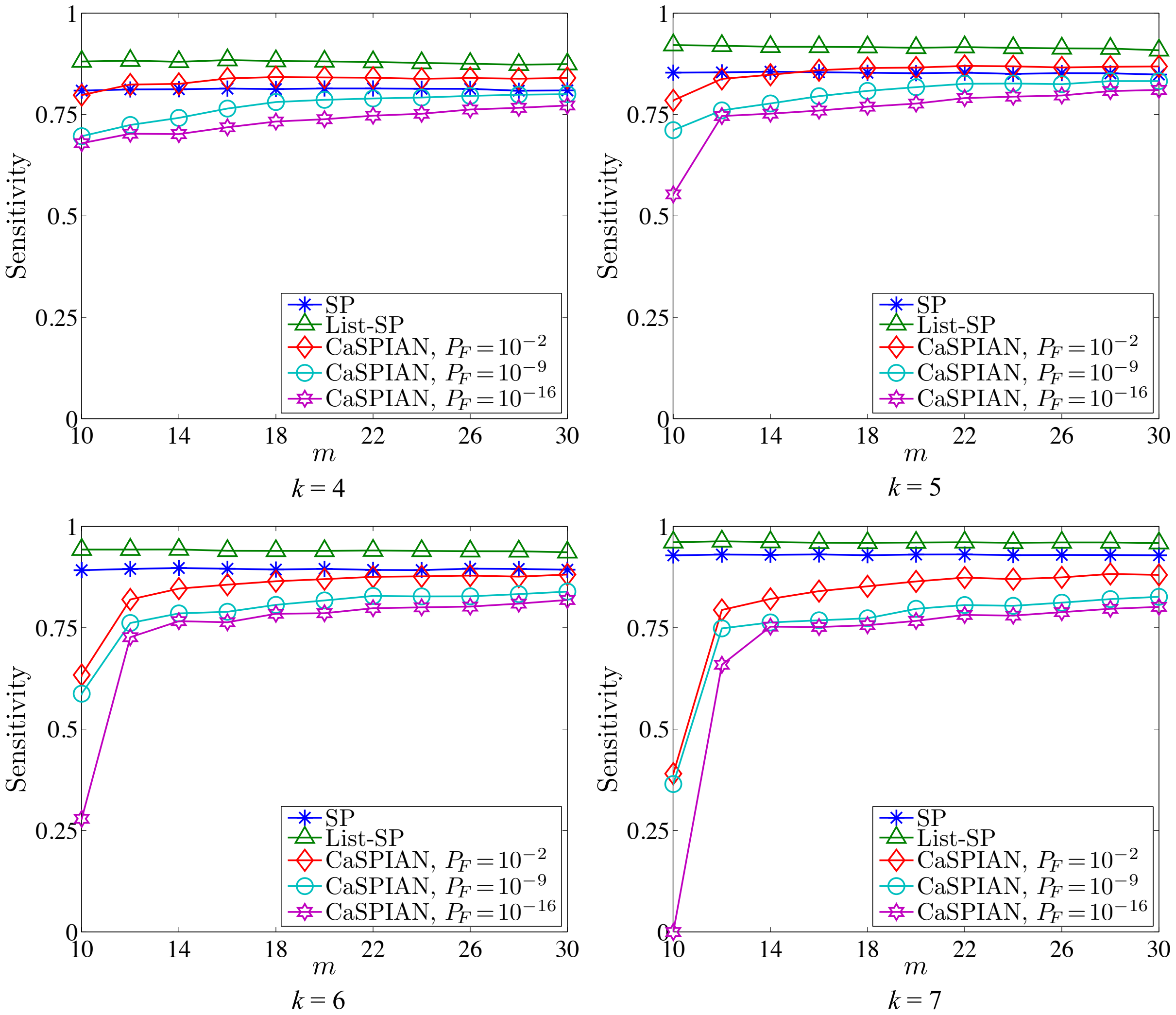

Figure S5. The average sensitivity of different algorithms for uniform gene expression model. The average sensitivity of different algorithms as a function of m, obtained from 200 randomly generated networks with $n = 10$. Uniform distribution was used to form the gene expressions.

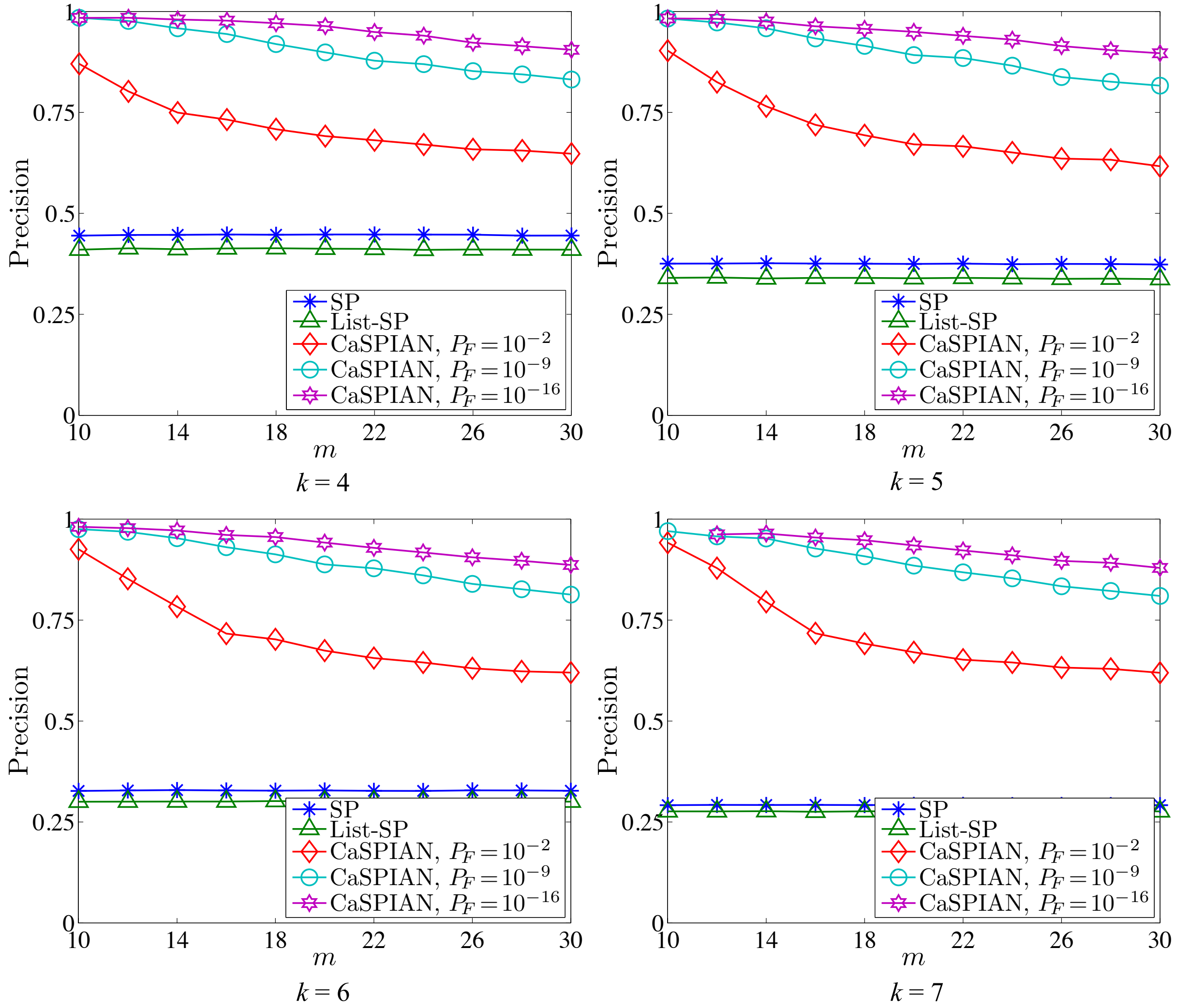

Figure S6. The average precision of different algorithms for uniform gene expression model. The average precision of different algorithms as a function of m, obtained from 200 randomly generated networks with $n = 10$. Uniform distribution was used to form the gene expressions.

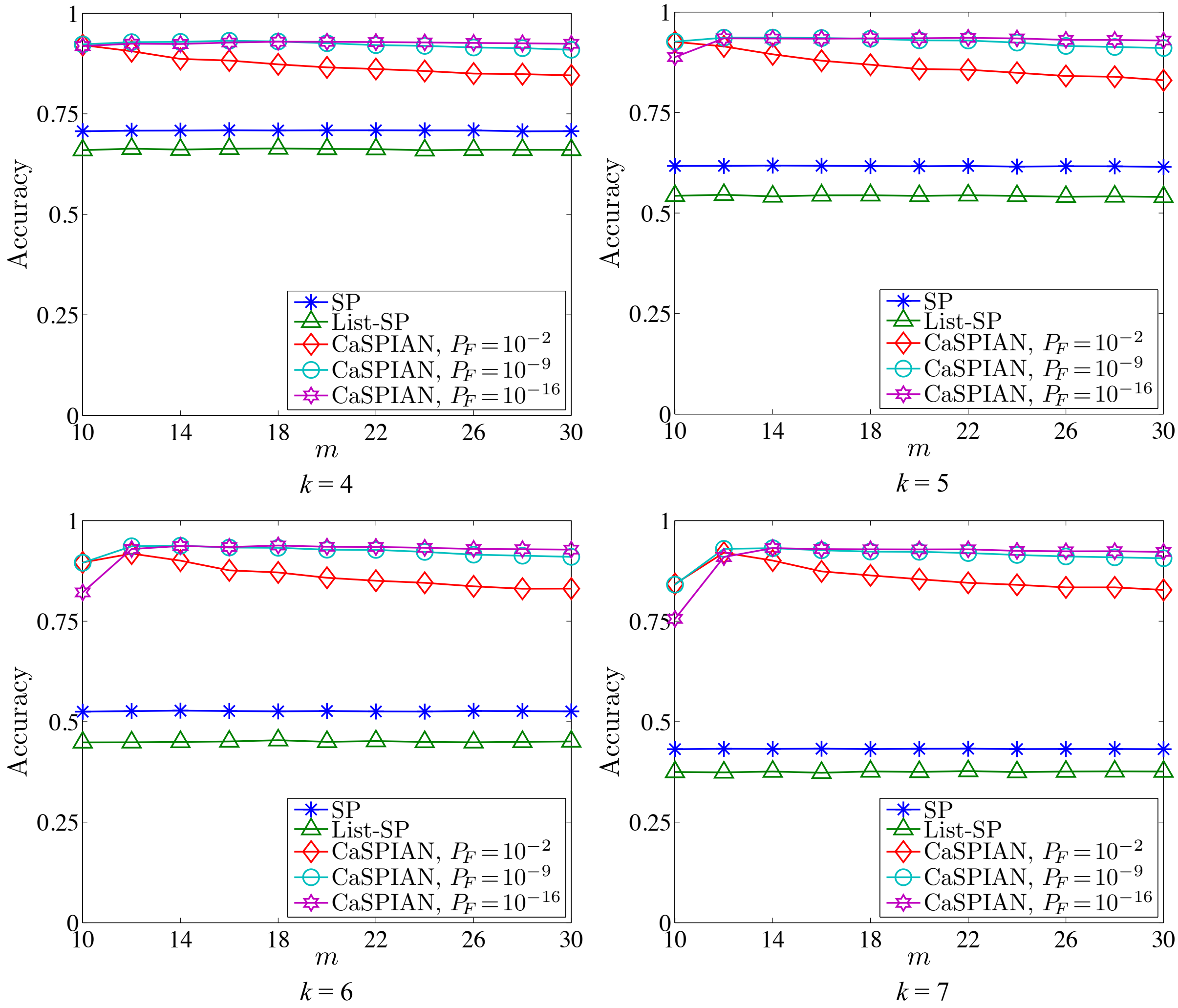

Figure S7. The average accuracy of different algorithms for uniform gene expression model. The average accuracy of different algorithms as a function of m, obtained from 200 randomly generated networks with $n = 10$. Uniform distribution was used to form the gene expressions.

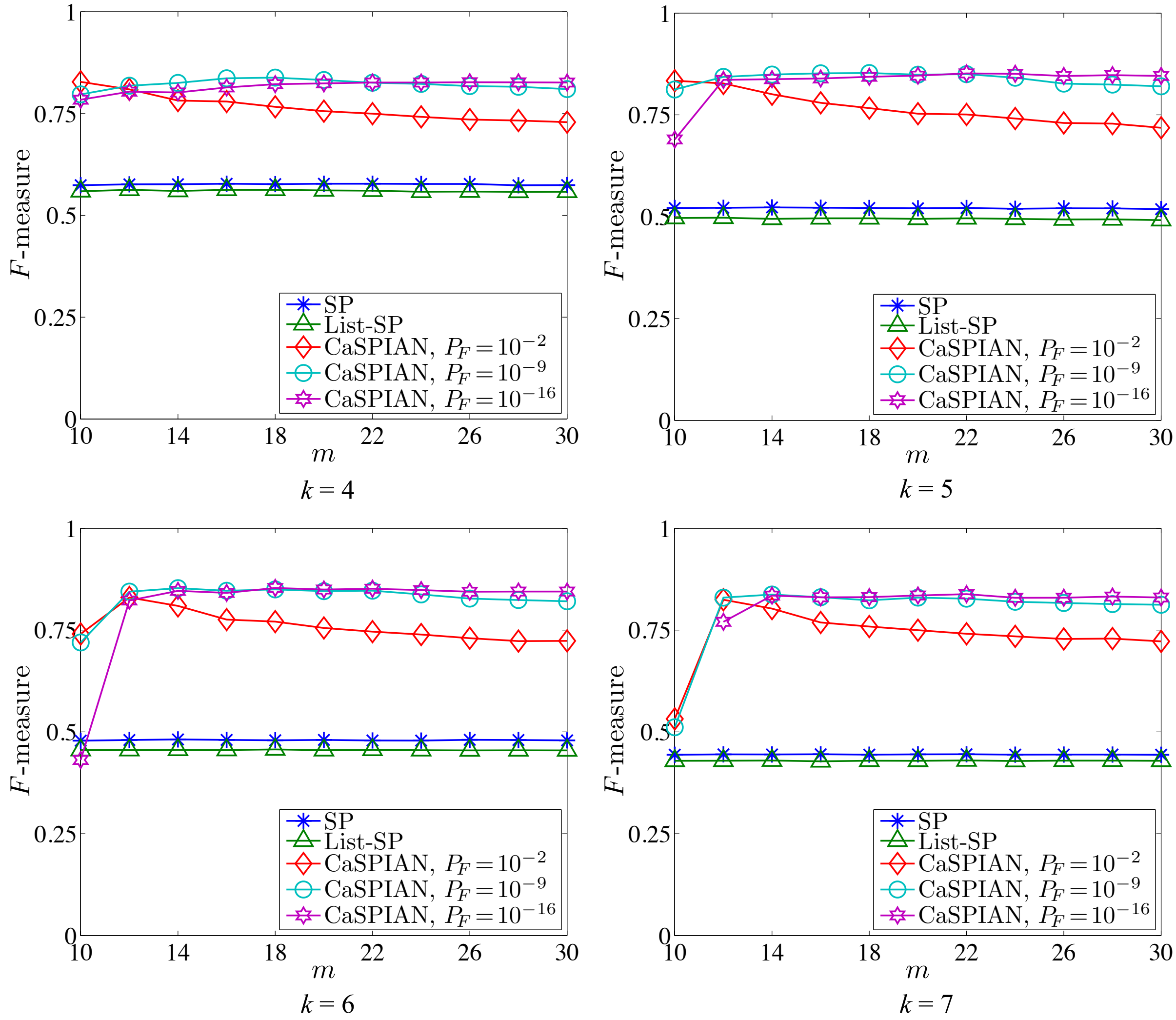

Figure S8. The average F-measure of different algorithms for uniform gene expression model. The average F-measure of different algorithms as a function of m, obtained from 200 randomly generated networks with $n = 10$. Uniform distribution was used to form the gene expressions.

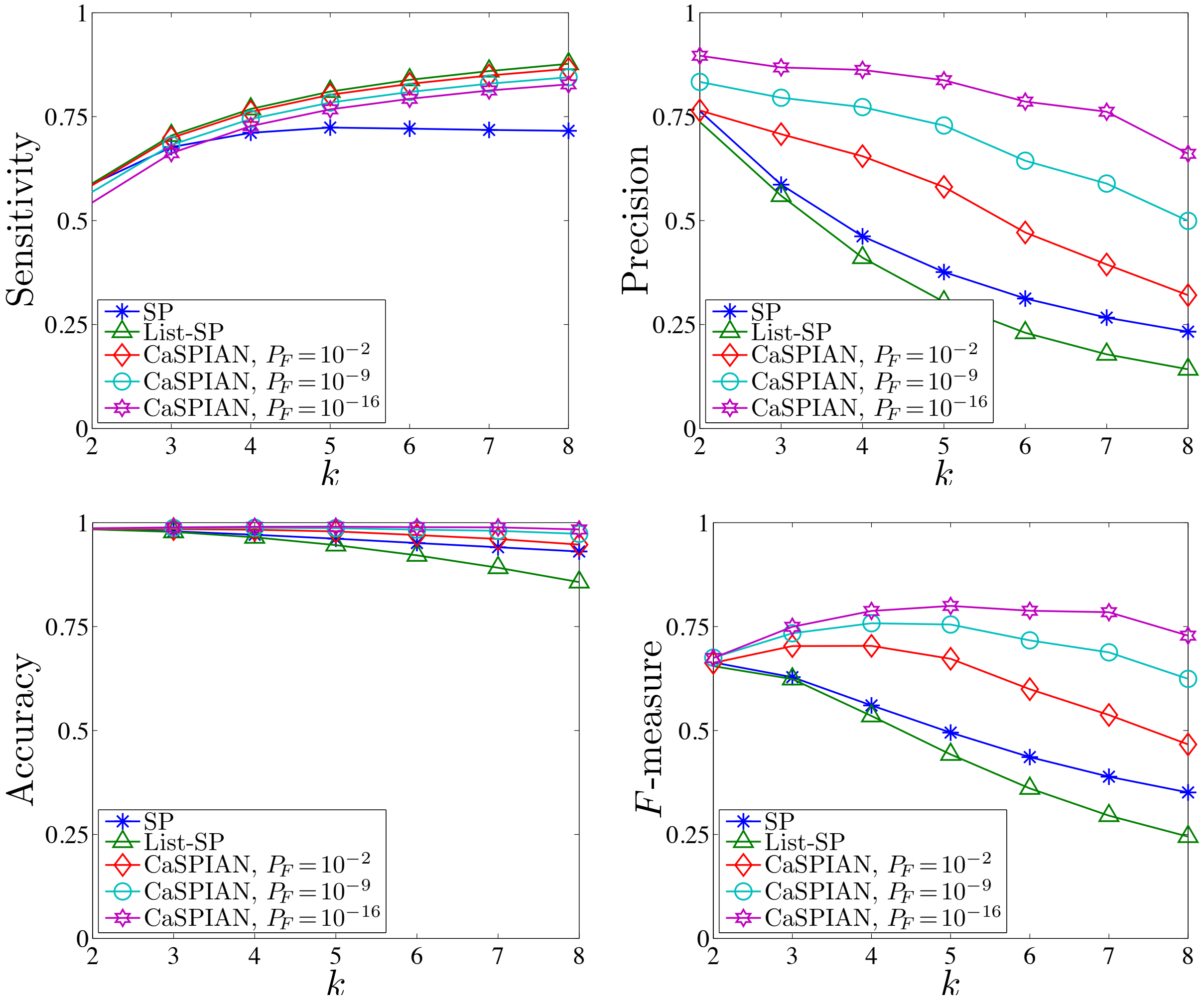

Figure S9. The average sensitivity, precision, accuracy, and F-measure of different algorithms as a function of sparsity. The results demonstrated are obtained from 500 randomly generated networks with $n = 100$, $m = L = 75$, $\gamma = 2.5$, $d_{min} = 2$, and $d_{\max} = 5$. Uniform distribution was used to form the gene expressions..

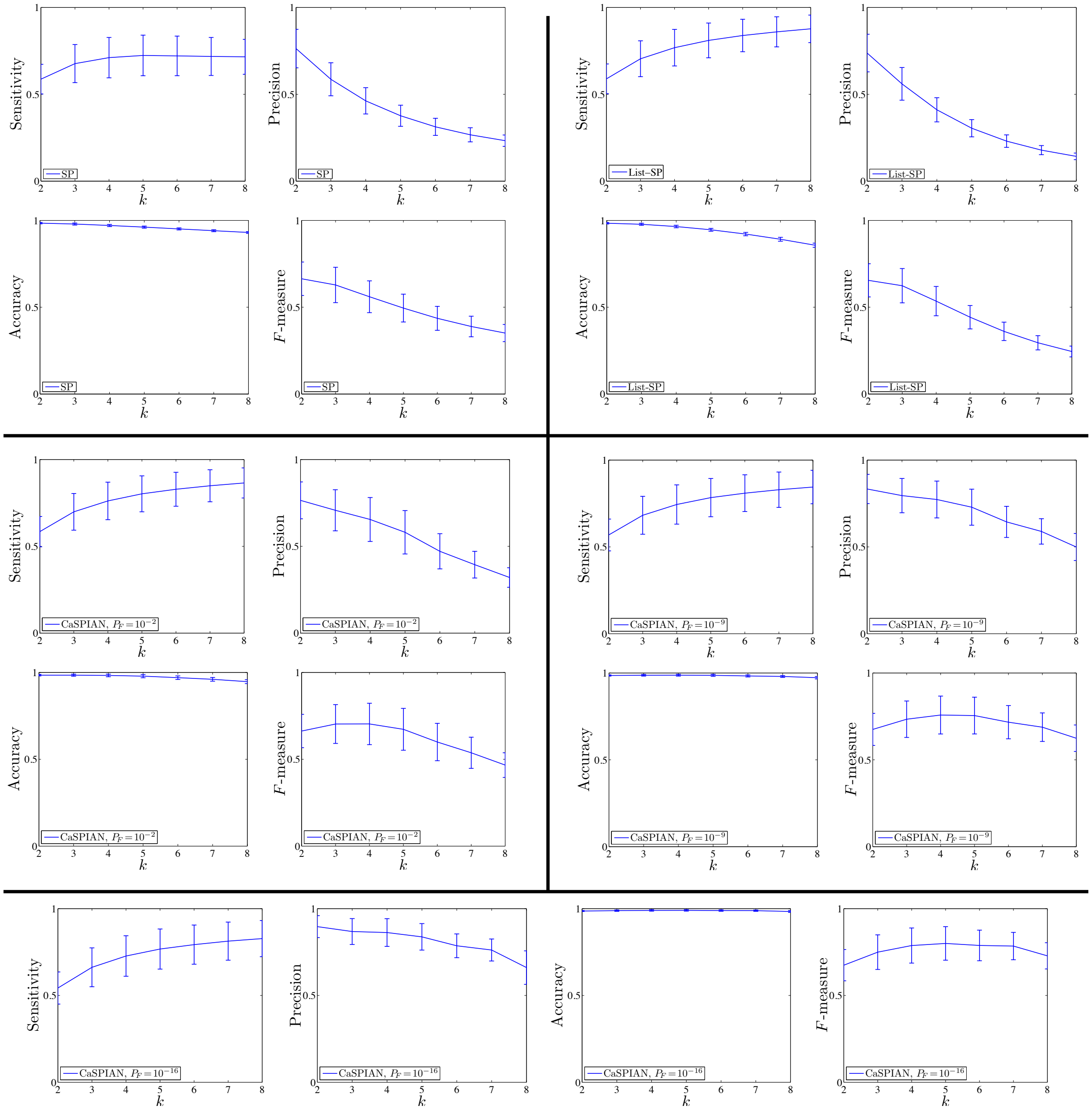

Figure S10. The average sensitivity, precision, accuracy, and F-measure, and the corresponding standard deviations for different algorithms as a function of sparsity. The demonstrated results are obtained from 500 randomly generated networks with $n = 100$, $m = L = 75$, $\gamma = 2.5$, $d_{min} = 2$, and $d_{\max} = 5$. Uniform distribution was used to form the gene expressions. The standard deviations are depicted using error bars.

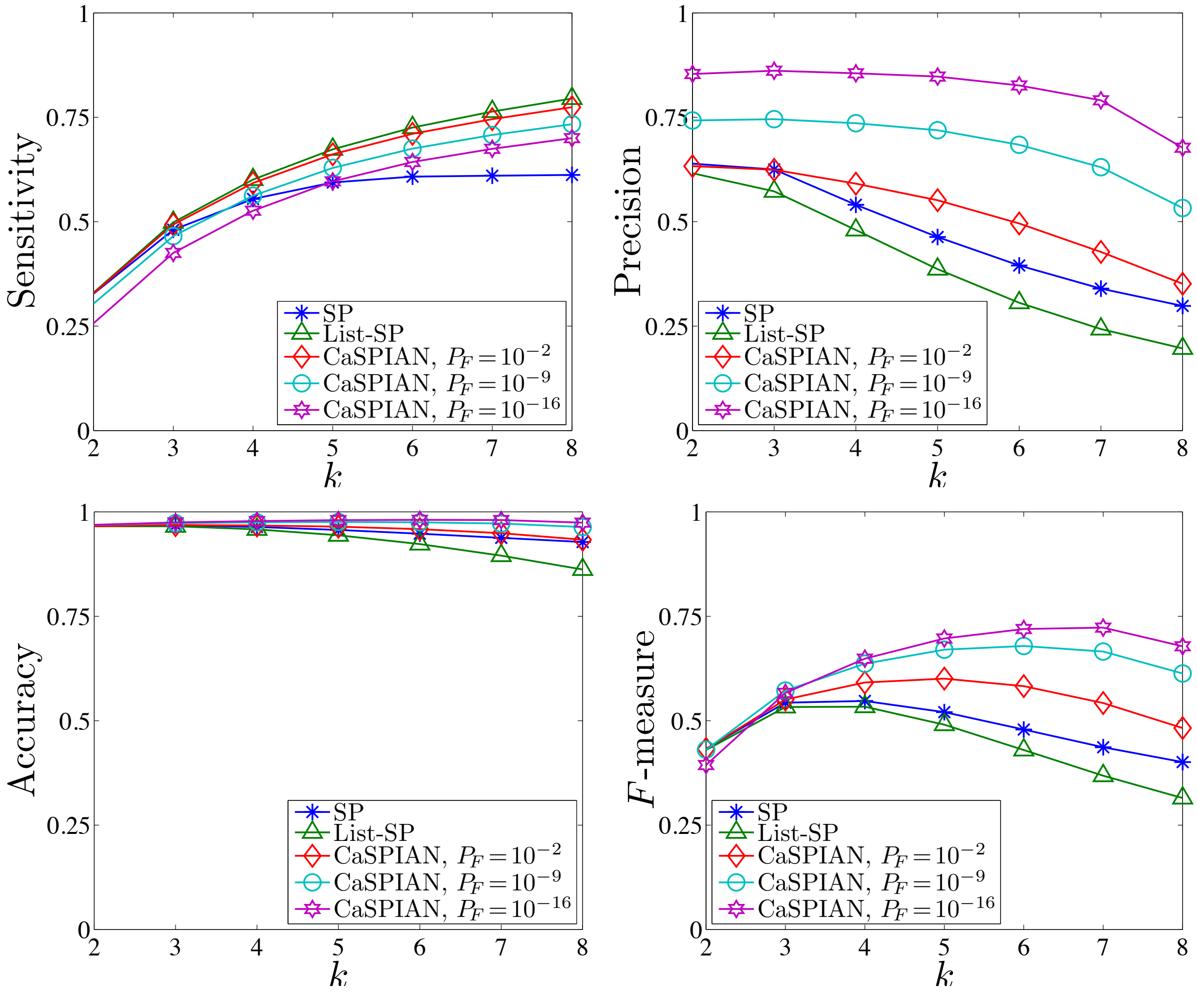

Figure S11. The average sensitivity, precision, accuracy, and F-measure of different algorithms as a function of sparsity. The demonstrated results are obtained from 500 randomly generated networks with $n = 100$, $m = L = 75$, $\gamma = 2$, $d_{min} = 3$, and $d_{\max} = 6$. Uniform distribution was used to form the gene expressions.

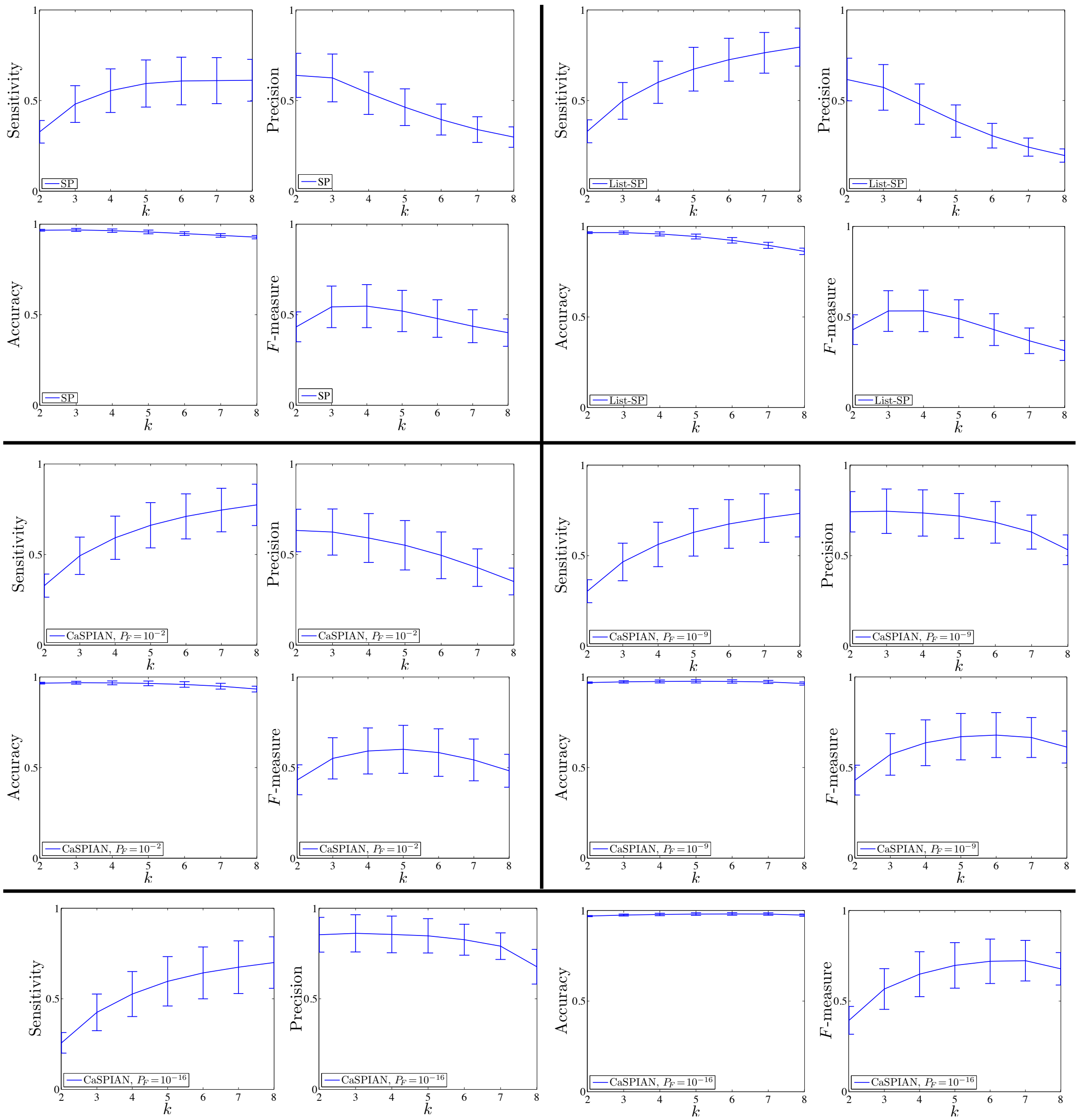

Figure S12. The average sensitivity, precision, accuracy, and F-measure, and the corresponding standard deviations for different algorithms as a function of sparsity. The demonstrated results are obtained from 500 randomly generated networks with $n = 100$, $m = L = 75$, $\gamma = 2$, $d_{min} = 3$, and $d_{\max} = 6$. Uniform distribution was used to form the gene expressions. The standard deviations are depicted using error bars.

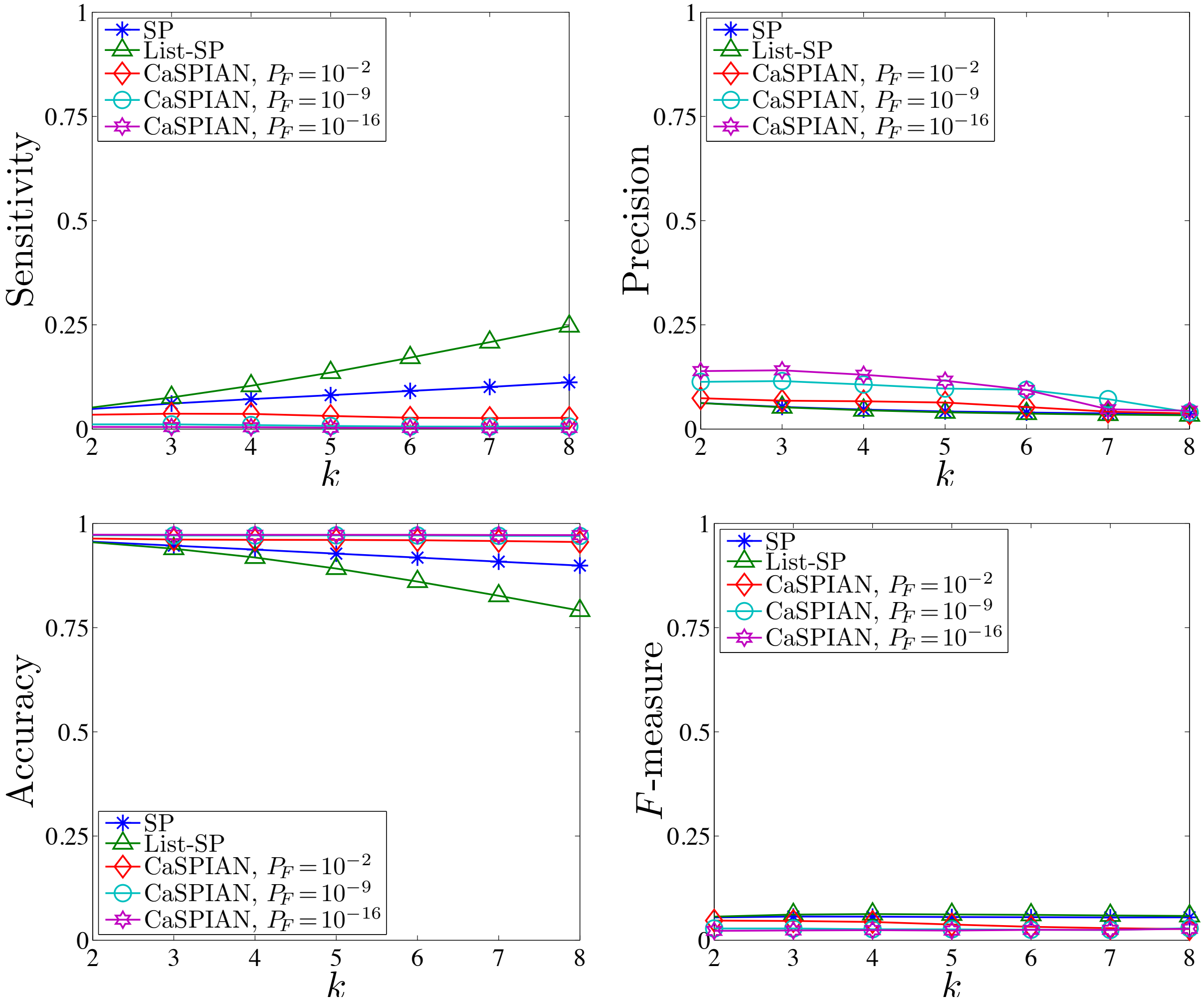

Figure S13. The average sensitivity, precision, accuracy, and F-measure of different algorithms as a function of sparsity for nonuniform sampling. The demonstrated results are obtained from 500 randomly generated networks with $n = 100$, $\gamma = 2.5$, $d_{min} = 2$, and $d_{\max} = 5$. Uniform distribution was used to form the gene expressions and an average sampling rate equal to 1/3 was used.

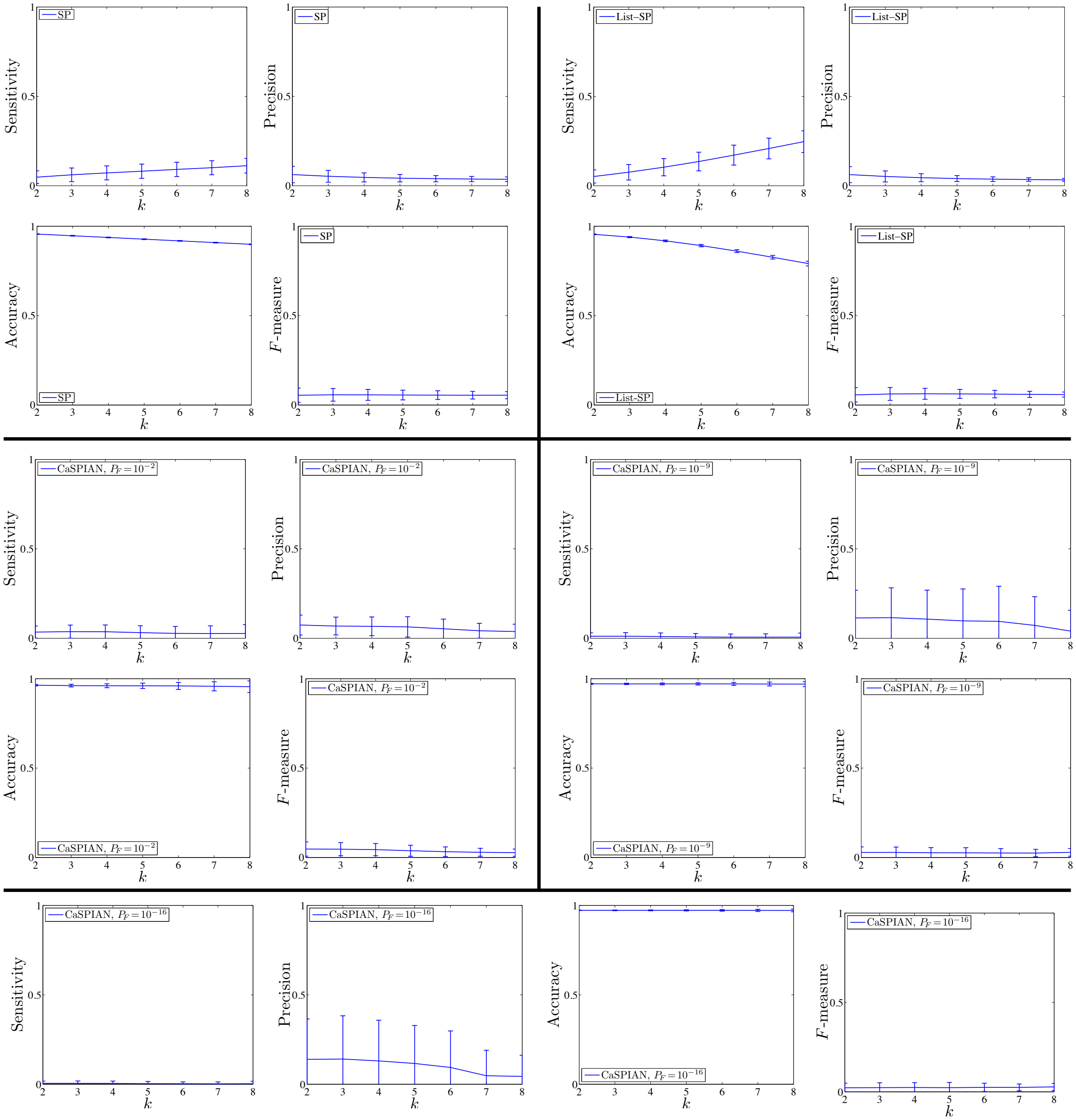

Figure S14. The average sensitivity, precision, accuracy, and F-measure of different algorithms and their corresponding standard deviations as a function of sparsity for nonuniform sampling. The demonstrated results are obtained from 500 randomly generated networks with $n = 100$, $\gamma = 2.5$, $d_{min} = 2$, and $d_{\max} = 5$. Uniform distribution was used to form the gene expressions and an average sampling rate equal to 1/3 was used.

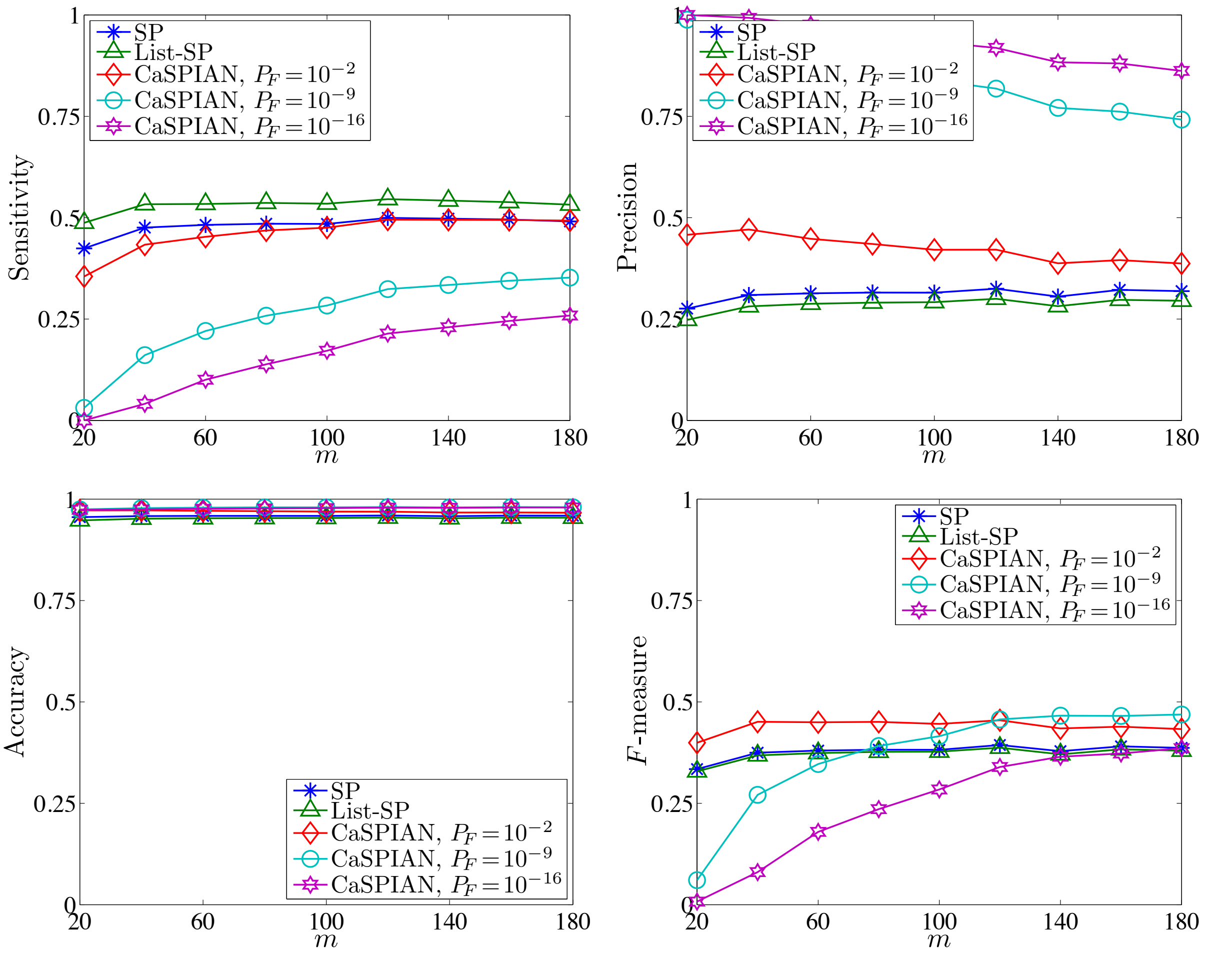

Figure S15. The average sensitivity, precision, accuracy, and F-measure of different algorithms as a function of m. The demonstrated results are obtained from 200 randomly generated networks with $n = 100$, $k = 4$, $\gamma = 2.5$, $d_{min} = 2$, and $d_{\max} = 5$. Uniform distribution was used to form the gene expressions and the ratio of the noise variance to signal power is equal to 5%.

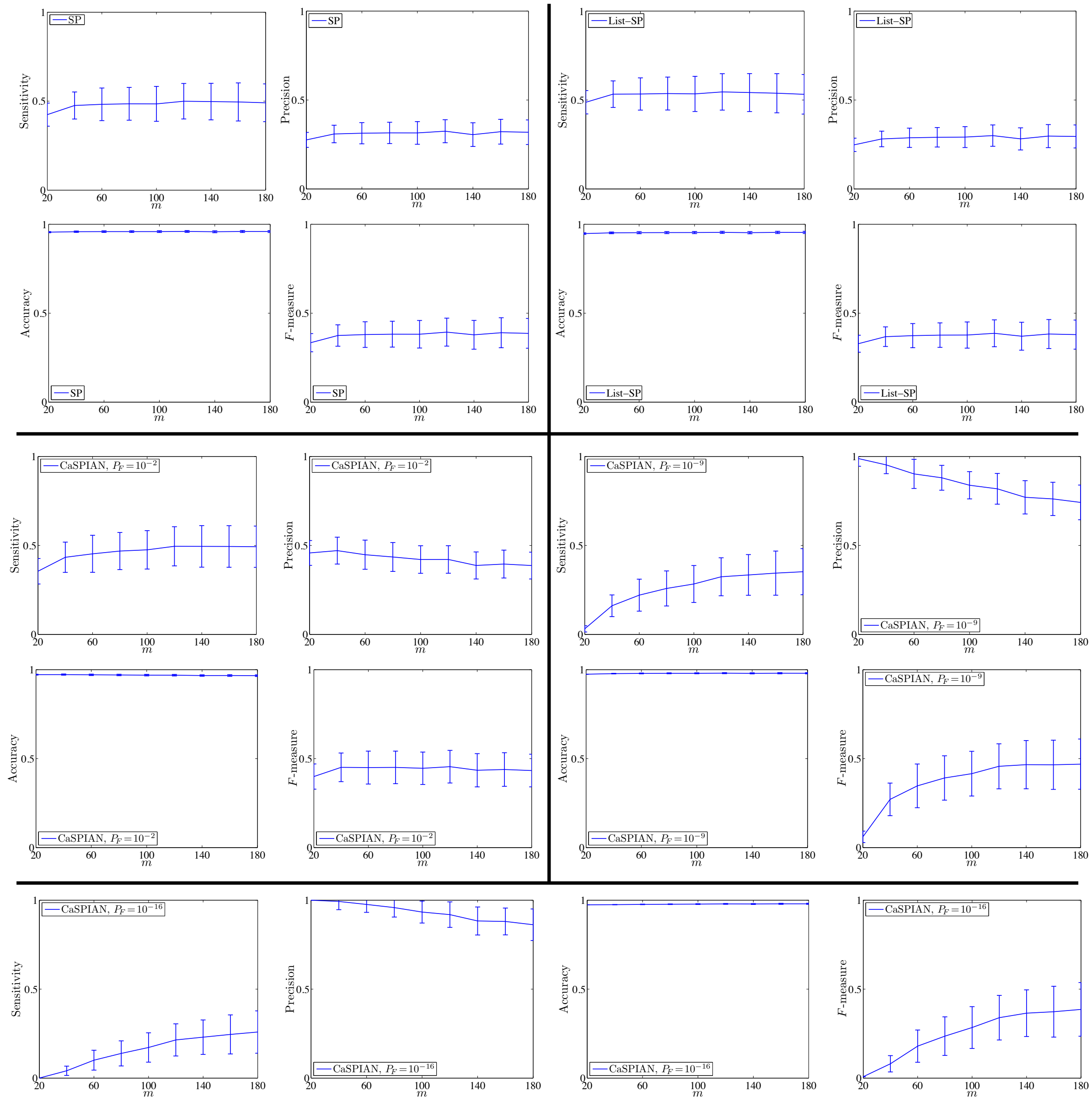

Figure S16. The average sensitivity, precision, accuracy, and F-measure, and their corresponding standard deviations for different algorithms as a function of m. The demonstrated results are obtained from 200 randomly generated networks with $n = 100$, $k = 4$, $\gamma = 2.5$, $d_{min} = 2$, and $d_{\max} = 5$. Uniform distribution was used to form the gene expressions and the ratio of the noise variance to signal power is equal to 5%. The standard deviations are depicted using error bars.

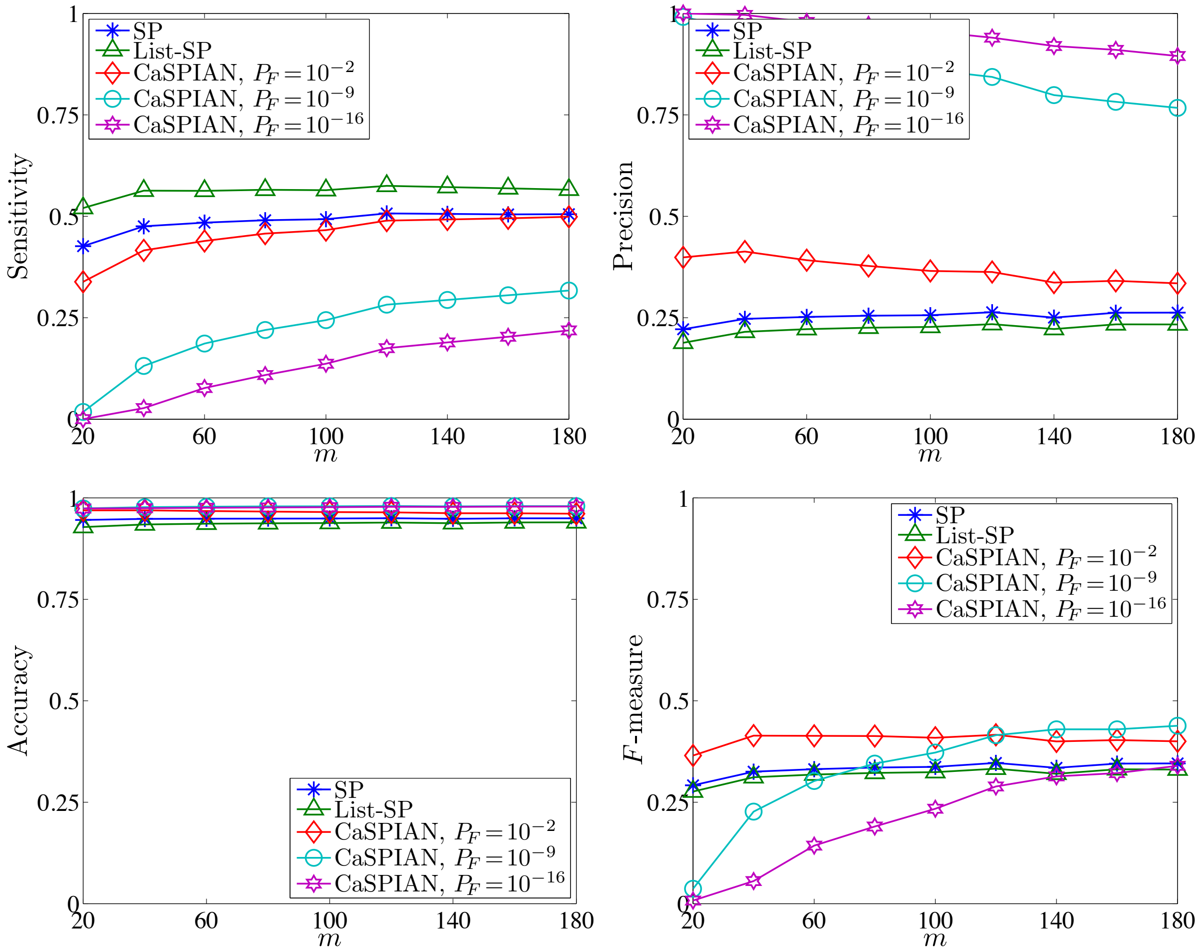

Figure S17. The average sensitivity, precision, accuracy, and F-measure of different algorithms as a function of m. The demonstrated results are obtained from 200 randomly generated networks with $n = 100$, $k = 5$, $\gamma = 2.5$, $d_{min} = 2$, and $d_{\max} = 5$. Uniform distribution was used to form the gene expressions and the ratio of the noise variance to signal power is equal to 5%.

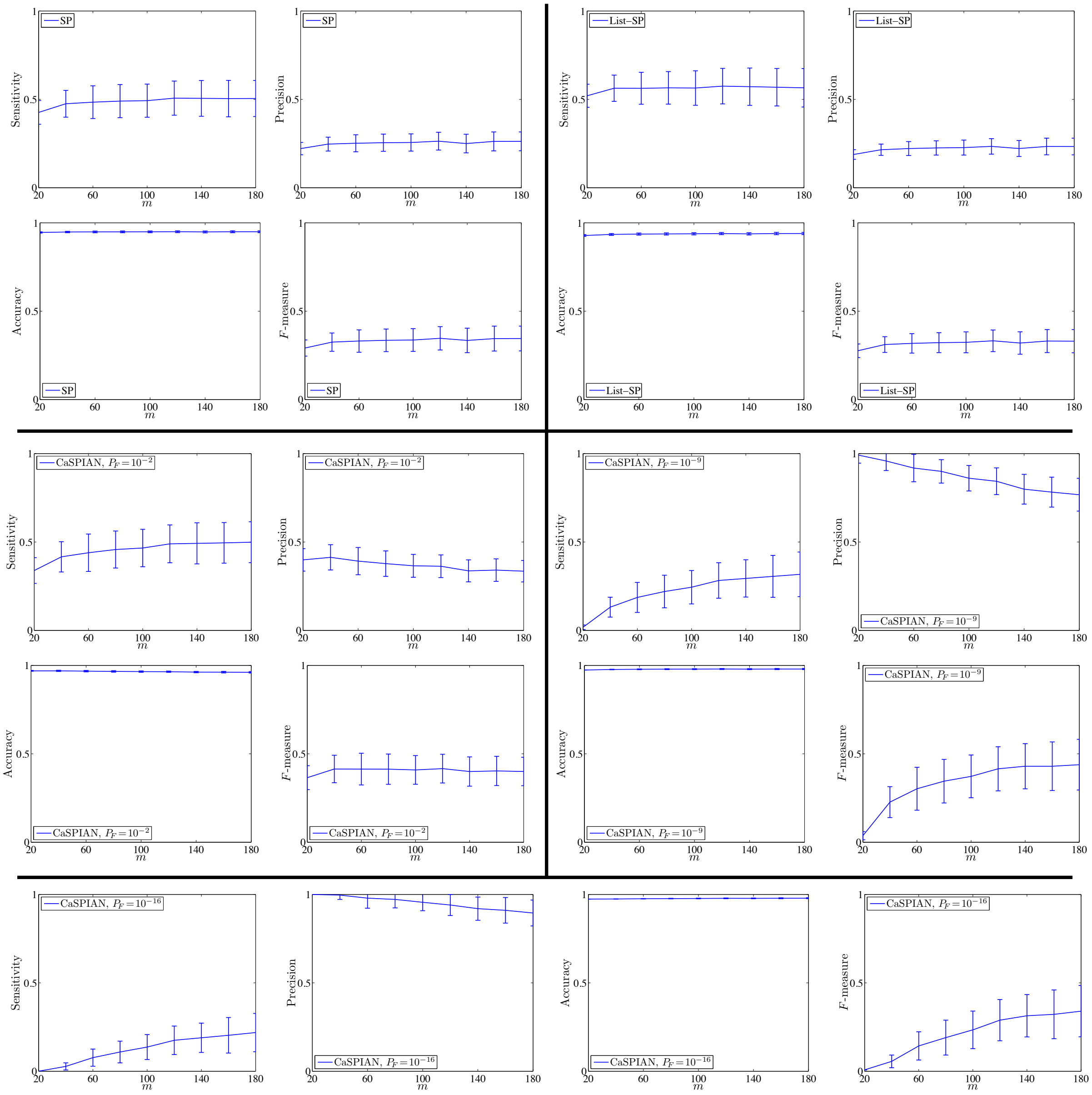

Figure S18. The average sensitivity, precision, accuracy, and F-measure, and their corresponding standard deviations for different algorithms as a function of m. The demonstrated results are obtained from 200 randomly generated networks with $n = 100$, $k = 5$, $\gamma = 2.5$, $d_{min} = 2$, and $d_{\max} = 5$. Uniform distribution was used to form the gene expressions and the ratio of the noise variance to signal power is equal to 5%. The standard deviations are depicted using error bars.

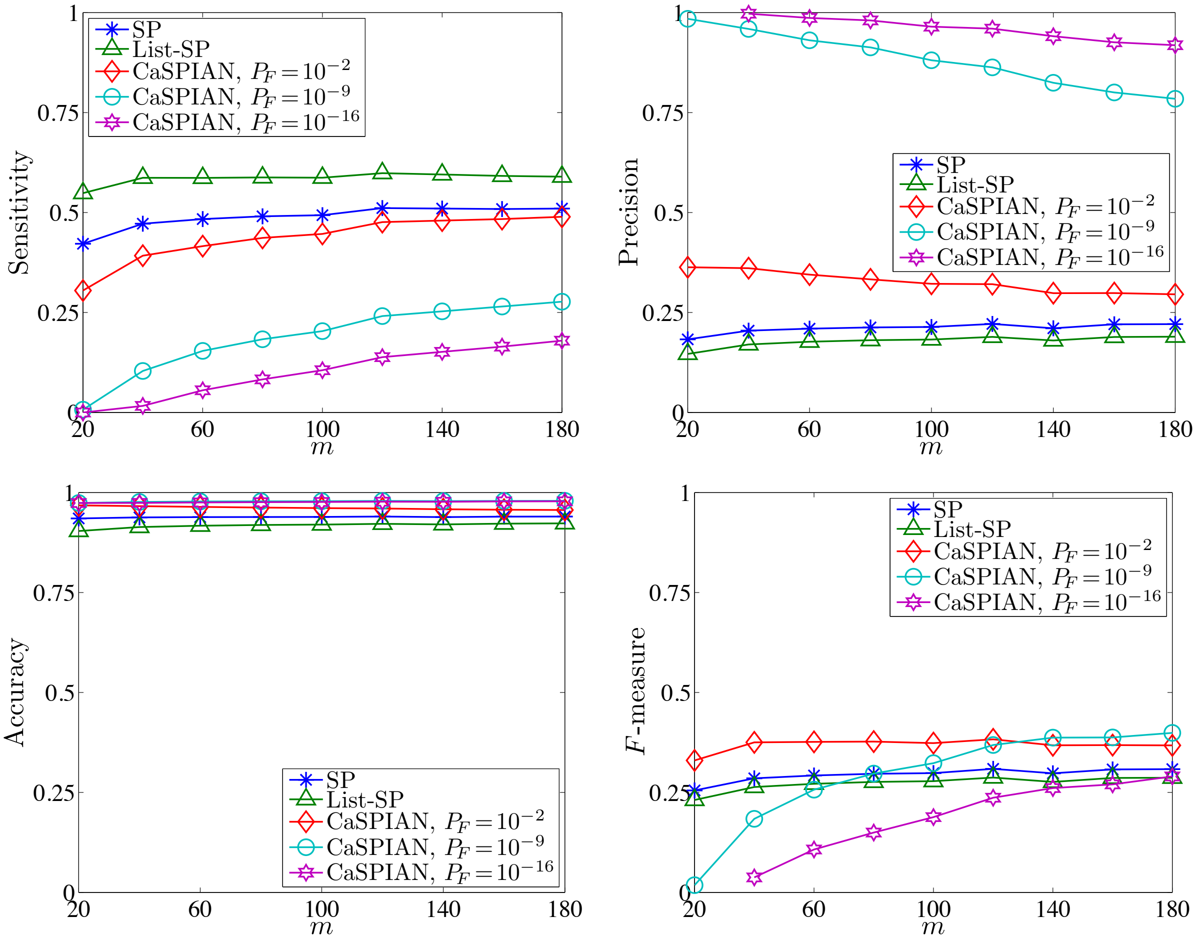

Figure S19. The average sensitivity, precision, accuracy, and F-measure of different algorithms as a function of m. The demonstrated results are obtained from 200 randomly generated networks with $n = 100$, $k = 6$, $\gamma = 2.5$, $d_{min} = 2$, and $d_{\max} = 5$. Uniform distribution was used to form the gene expressions and the ratio of the noise variance to signal power is equal to 5%.

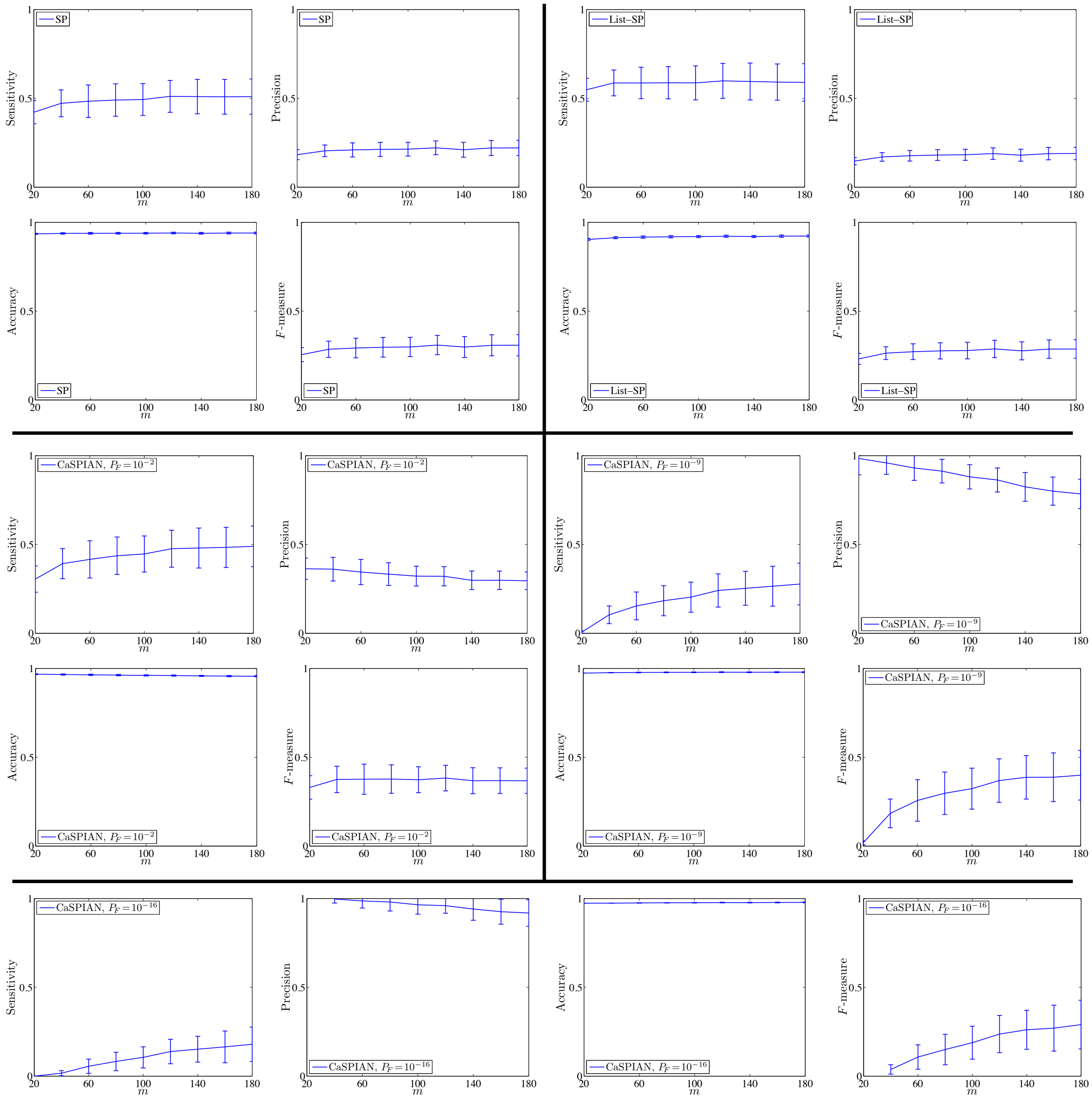

Figure S20. The average sensitivity, precision, accuracy, and F-measure, and their corresponding standard deviations for different algorithms as a function of m. The demonstrated results are obtained from 200 randomly generated networks with $n = 100$, $k = 6$, $\gamma = 2.5$, $d_{min} = 2$, and $d_{\max} = 5$. Uniform distribution was used to form the gene expressions and the ratio of the noise variance to signal power is equal to 5%. The standard deviations are depicted using error bars.

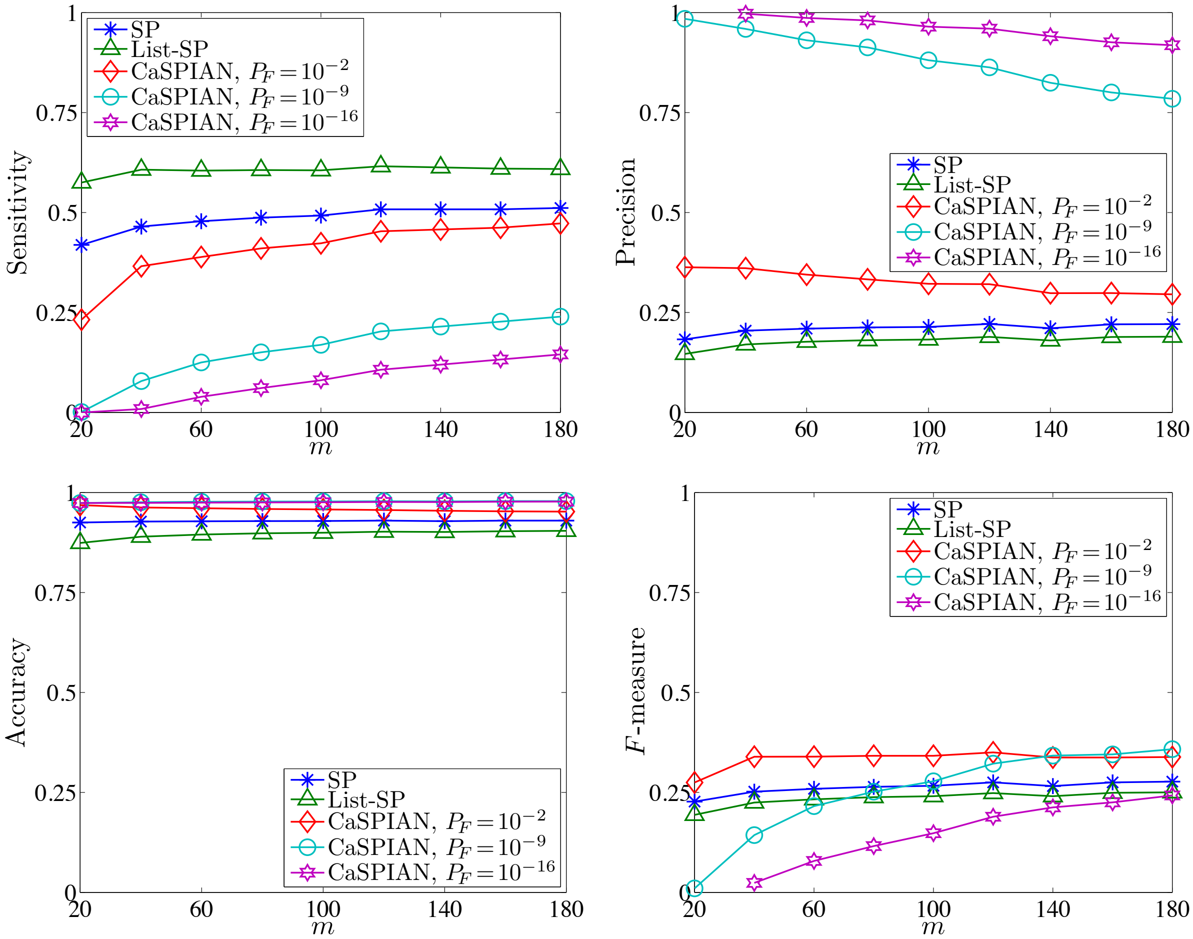

Figure S21. The average sensitivity, precision, accuracy, and F-measure of different algorithms as a function of m. The demonstrated results are obtained from 200 randomly generated networks with $n = 100$, $k = 7$, $\gamma = 2.5$, $d_{min} = 2$, and $d_{\max} = 5$. Uniform distribution was used to form the gene expressions and the ratio of the noise variance to signal power is equal to 5%.

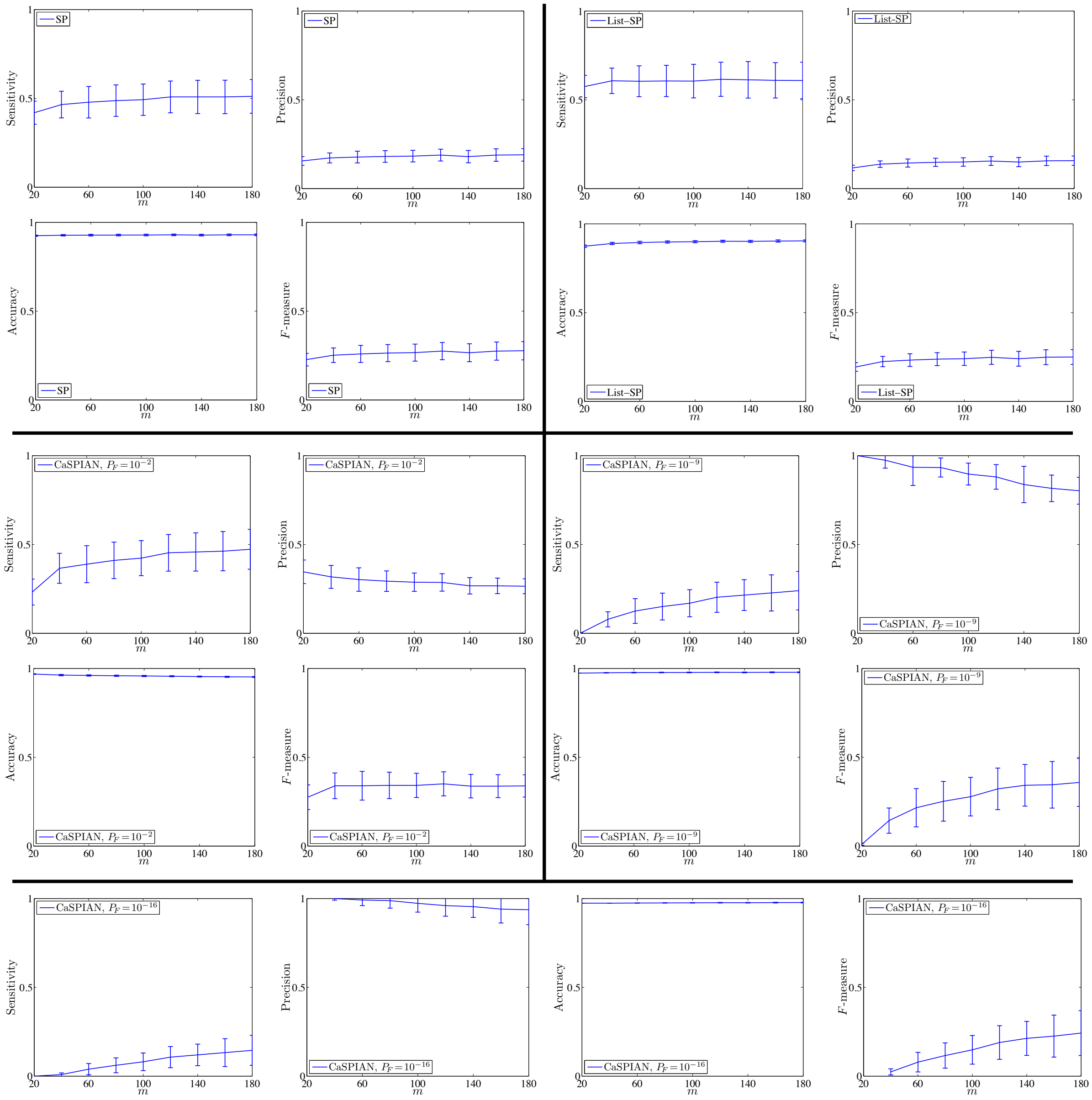

Figure S22. The average sensitivity, precision, accuracy, and F-measure, and their corresponding standard deviations for different algorithms as a function of m. The demonstrated results are obtained from 200 randomly generated networks with $n = 100$, $k = 7$, $\gamma = 2.5$, $d_{min} = 2$, and $d_{\max} = 5$. Uniform distribution was used to form the gene expressions and the ratio of the noise variance to signal power is equal to 5%. The standard deviations are depicted using error bars.

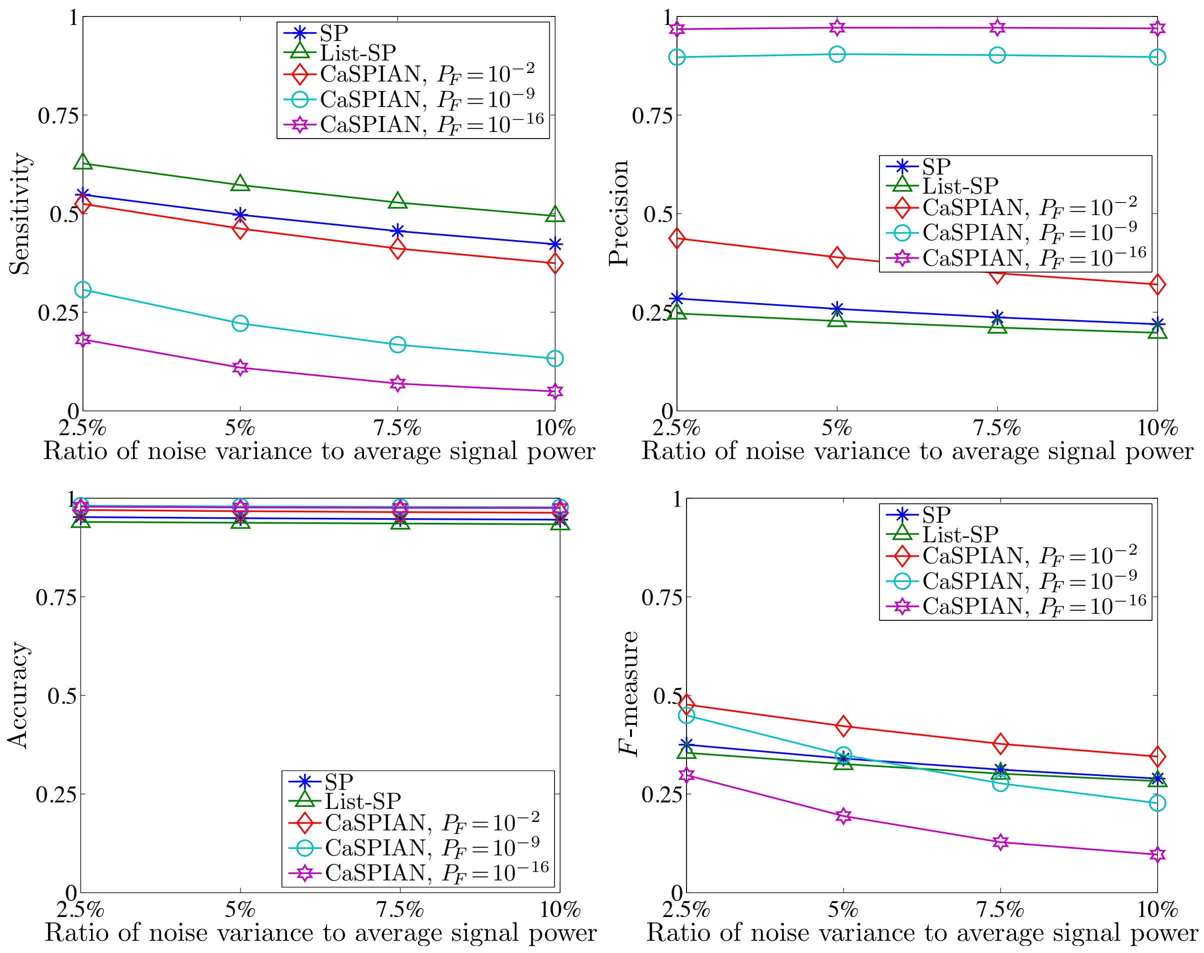

Figure S23. The average sensitivity, precision, accuracy, and F-measure of different algorithms for various noise levels. The demonstrated results are obtained from 500 randomly generated networks with $n = 100$, $m = L = 75$, $\gamma = 2.5$, $d_{min} = 2$, and $d_{\max} = 5$. Uniform distribution was used to form the gene expressions.

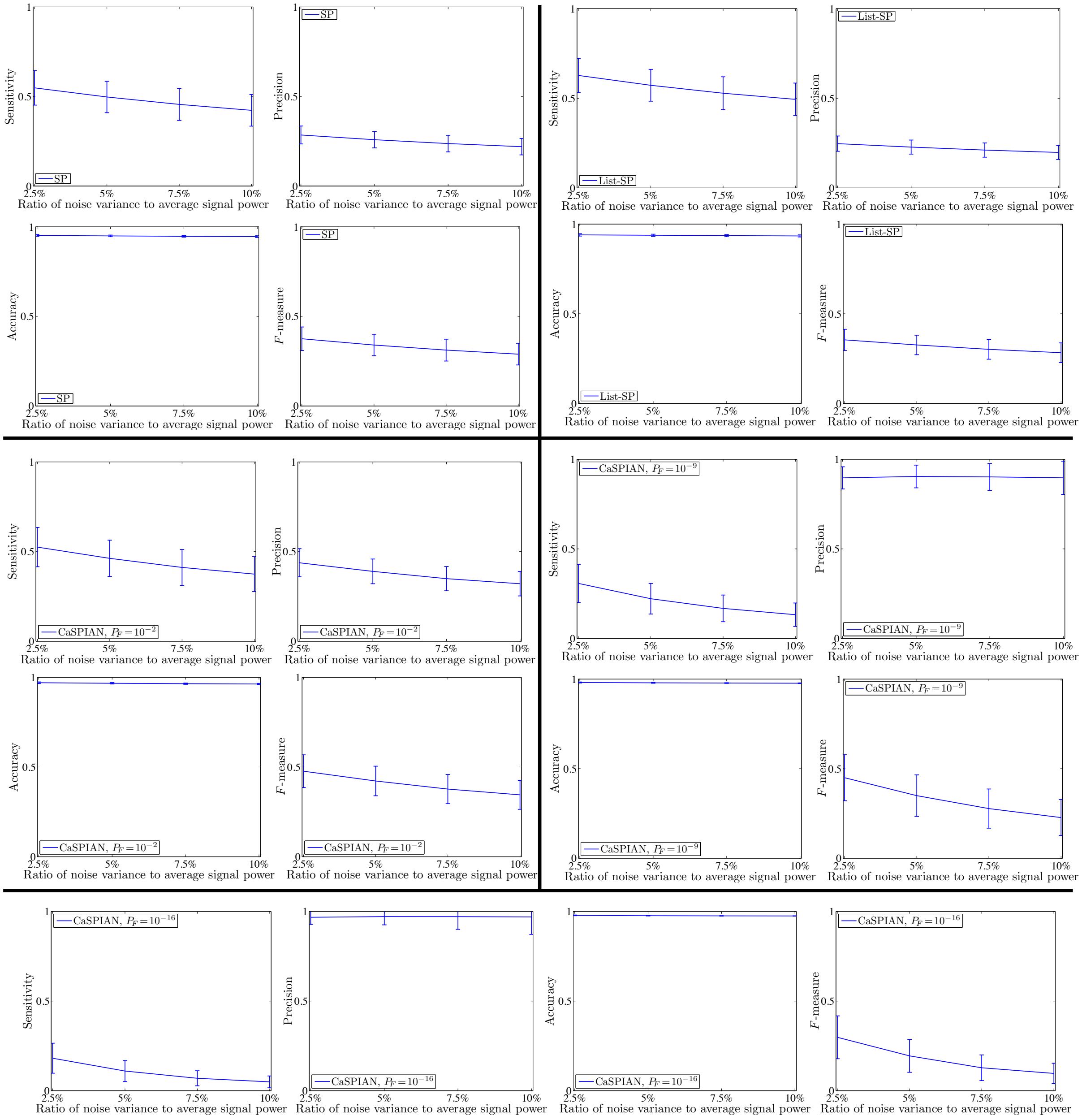

Figure S24. The average sensitivity, precision, accuracy, and F-measure of different algorithms with their corresponding standard deviation for various noise levels. The demonstrated results are obtained from 500 randomly generated networks with $n = 100$, $m = L = 75$, $\gamma = 2.5$, $d_{min} = 2$, and $d_{\max} = 5$. Uniform distribution was used to form the gene expressions.
