# Supplementary material for: CaSPIAN: A Causal Compressive Sensing Algorithm for Discovering Directed Interactions in Gene Networks
Source: PLoS One. 2014 Mar 12;9(3):e90781. doi: 10.1371/journal.pone.0090781 (PMC3951243; doi:10.1371/journal.pone.0090781)
Supplement: Appendix S2 — Tables of multiplicity ratios (MRs) for the E. coli SOS network. (PDF) [file pone.0090781.s002.pdf]

Appendix S2: Tables of multiplicity ratios (MRs) for the *E. coli* SOS network

Table S1. The adjacency matrix of the SOS network according to Table S4 of [1]. A value 1 in the entry (i, j) of the table corresponds to a link from the gene indexed by j to the gene indexed by i. A value of 0 means that no link was reported in that direction; self links (i.e. loops) are not shown.

	dinI	lexA	recA	recF	rpoD	rpoH	rpoS	ssb	umuC/D
dinI		1	1	1	1	0	0	1	1
lexA	1		1	1	1	0	0	1	1
recA	1	1		1	1	0	0	1	1
recF	0	0	0		1	0	1	0	0
rpoD	1	1	1	1		1	0	1	1
rpoH	0	0	0	0	1		0	0	0
rpoS	0	0	0	0	1	0		0	0
ssb	1	1	1	1	1	0	0		1
umuC/D	1	1	1	1	1	0	0	1	

Table S2. The MR for different links in the SOS network inferred using List-SP for $k = 4$. A value 1 in the entry (i, j) of the table corresponds to a link from the gene indexed by j to the gene indexed by i. A value of 0 shows that no link was found in that direction, in any run of the algorithm.

	dinI	lexA	recA	recF	rpoD	rpoH	rpoS	ssb	umuC/D
dinI		1	0.7	0	0	0	0	0	1
lexA	1		1	0	0	0	0	0	0.4
recA	0.8	1		0	0	0	0	0	0.7
recF	0.1	0	0.1		1	0	0	0	0.6
rpoD	0	0	0	1		1	0	0	0
rpoH	0	0	0	0	1		0	0	0
rpoS	0	0	0	0	0	0		0.1	0
ssb	1	0.2	0	0	0.1	0	0		1
umuC/D	1	1	0.4	0	0	0	0	0	

Table S3. The MR for different links in the SOS network inferred using List-SP for $k = 5$. A value 1 in the entry (i, j) of the table corresponds to a link from the gene indexed by j to the gene indexed by i. A value of 0 shows that no link was found in that direction, in any run of the algorithm.

	dinI	lexA	recA	recF	rpoD	rpoH	rpoS	ssb	umuC/D
dinI		1	0.8	0	0	0	0	0	1
lexA	1		1	0	0	0.1	0	0	0.7
recA	0.9	1		0	0.1	0	0	0	0.9
recF	0.1	0	0.1		1	0	0	0	0.6
rpoD	0.1	0	0	1		1	0	0	0.1
rpoH	0	0	0	0	1		0	0	0
rpoS	0.1	0	0	0	0.1	0		0.1	0.1
ssb	1	0.3	0	0	0.1	0	0		1
umuC/D	1	1	0.7	0	0	0	0	0	

Table S4. The MR for different links in the SOS network inferred using List-SP for $k = 6$. A value 1 in the entry (i, j) of the table corresponds to a link from the gene indexed by j to the gene indexed by i. A value of 0 shows that no link was found in that direction, in any run of the algorithm.

	dinI	lexA	recA	recF	rpoD	rpoH	rpoS	ssb	umuC/D
dinI		1	0.9	0	0	0	0	0	1
lexA	1		1	0	0	0.1	0	0	0.9
recA	1	1		0	0.5	0	0	0	1
recF	0.2	0	0.1		1	0	0	0	0.9
rpoD	0.3	0.1	0	1		1	0	0	0.1
rpoH	0.1	0	0	0	1		0	0	0
rpoS	0.1	0	0	0	0.2	0		0.2	0.1
ssb	1	0.5	0.3	0	0.2	0	0		1
umuC/D	1	1	0.7	0	0	0	0	0	

Table S5. The MR for different links in the SOS network inferred using CaSPIAN for $k = 5$ and $P_F = 10^{-2}$. A value 1 in the entry (i, j) of the table corresponds to a link from the gene indexed by j to the gene indexed by i. A value of 0 shows that no link was found in that direction, in any run of the algorithm.

	dinI	lexA	recA	recF	rpoD	rpoH	rpoS	ssb	umuC/D
dinI		0.8	0.5	0	0	0	0	0	0.2
lexA	0.8		0.5	0	0	0	0	0	0
recA	0.2	0.4		0	0.1	0	0	0	0
recF	0	0	0.1		1	0	0	0	0.1
rpoD	0	0	0	0.9		1	0	0	0
rpoH	0	0	0	0	0.9		0	0	0
rpoS	0.1	0	0	0	0.1	0		0.1	0
ssb	0.1	0	0	0	0.1	0	0		0.2
umuC/D	0.3	0.1	0	0	0	0	0	0	

Table S6. The MR for different links in the SOS network inferred using CaSPIAN for $k = 5$ and $P_F = 10^{-4}$. A value 1 in the entry (i, j) of the table corresponds to a link from the gene indexed by j to the gene indexed by i. A value of 0 shows that no link was found in that direction, in any run of the algorithm.

	dinI	lexA	recA	recF	rpoD	rpoH	rpoS	ssb	umuC/D
dinI		0.1	0	0	0	0	0	0	0
lexA	0.4		0.2	0	0	0	0	0	0
recA	0	0.3		0	0	0	0	0	0
recF	0	0	0		0.9	0	0	0	0
rpoD	0	0	0	0.2		0.9	0	0	0
rpoH	0	0	0	0	0.5		0	0	0
rpoS	0	0	0	0	0	0		0	0
ssb	0	0	0	0	0	0	0		0
umuC/D	0.1	0	0	0	0	0	0	0	

Table S7. The MR for different links in the SOS network inferred using CaSPIAN for $k = 5$ and $P_F = 10^{-6}$. A value 1 in the entry (i, j) of the table corresponds to a link from the gene indexed by j to the gene indexed by i. A value of 0 shows that no link was found in that direction, in any run of the algorithm.

	dinI	lexA	recA	recF	rpoD	rpoH	rpoS	ssb	umuC/D
dinI		0.1	0	0	0	0	0	0	0
lexA	0.1		0	0	0	0	0	0	0
recA	0	0.2		0	0	0	0	0	0
recF	0	0	0		0.3	0	0	0	0
rpoD	0	0	0	0		0.5	0	0	0
rpoH	0	0	0	0	0.4		0	0	0
rpoS	0	0	0	0	0	0		0	0
ssb	0	0	0	0	0	0	0		0
umuC/D	0	0	0	0	0	0	0	0	

Table S8. The MR for different links in the SOS network inferred using CaSPIAN for $k = 5$ and $P_F = 10^{-8}$. A value 1 in the entry (i, j) of the table corresponds to a link from the gene indexed by j to the gene indexed by i. A value of 0 shows that no link was found in that direction, in any run of the algorithm.

	dinI	lexA	recA	recF	rpoD	rpoH	rpoS	ssb	umuC/D
dinI		0	0	0	0	0	0	0	0
lexA	0.1		0	0	0	0	0	0	0
recA	0	0		0	0	0	0	0	0
recF	0	0	0		0.2	0	0	0	0
rpoD	0	0	0	0		0.5	0	0	0
rpoH	0	0	0	0	0.2		0	0	0
rpoS	0	0	0	0	0	0		0	0
ssb	0	0	0	0	0	0	0		0
umuC/D	0	0	0	0	0	0	0	0	

Table S9. The MR for different links in the SOS network inferred using CaSPIAN for $k = 5$ and $P_F = 10^{-10}$. A value 1 in the entry (i, j) of the table corresponds to a link from the gene indexed by j to the gene indexed by i. A value of 0 shows that no link was found in that direction, in any run of the algorithm.

	dinI	lexA	recA	recF	rpoD	rpoH	rpoS	ssb	umuC/D
dinI		0	0	0	0	0	0	0	0
lexA	0		0	0	0	0	0	0	0
recA	0	0		0	0	0	0	0	0
recF	0	0	0		0	0	0	0	0
rpoD	0	0	0	0		0.2	0	0	0
rpoH	0	0	0	0	0		0	0	0
rpoS	0	0	0	0	0	0		0	0
ssb	0	0	0	0	0	0	0		0
umuC/D	0	0	0	0	0	0	0	0	

References

1. Gardner TS, di Bernardo D, Lorenz D, Collins JJ (2003) Inferring genetic networks and identifying compound mode of action via expression profiling. Science 301: 102-105.
